# Event-related potential (ERP) correlates of face processing in verbal children with autism spectrum disorders (ASD) and their first-degree relatives: a family study

**DOI:** 10.1186/s13229-018-0220-x

**Published:** 2018-07-05

**Authors:** Olga V. Sysoeva, John N. Constantino, Andrey P. Anokhin

**Affiliations:** 1 Washington University School of Medicine, 660 South Euclid Avenue, Campus Box 8504, Saint Louis, MO USA; 2 Autism Research Laboratory, Moscow State University of Psychology and Education (MSUPE), 2A Shelepihinskaya Quay, Moscow, 123390 Russia

**Keywords:** Autistic disorder, Electrophysiology, ERP, N170, Endophenotype

## Abstract

**Background:**

Inherited abnormalities of perception, recognition, and attention to faces have been implicated in the etiology of autism spectrum disorders (ASD) including abnormal components of event-related brain potentials (ERP) elicited by faces.

**Methods:**

We examined familial aggregation of face processing ERP abnormalities previously implicated in ASD in 49 verbal individuals with ASD, 36 unaffected siblings (US), 18 unaffected fathers (UF), and 53 unrelated controls (UC). The ASD, US, and UC groups ranged in age from 12 to 21 years, the UF group ranged in age from 30 to 56 years. ERP responses to images of upright and inverted faces and houses were analyzed under disparate EEG reference schemes.

**Results:**

Face-sensitive features of N170 and P1 were readily observed in all groups. Differences between ASD and control groups depended upon the EEG reference scheme. Notably, the superiority of face over object for N170 latency was attenuated in ASD subjects, but not their relatives; this occurred exclusively with the average reference. The difference in N170 amplitude between inverted and upright faces was reduced in both ASD and US groups relative to UC, but this effect was significant only with the vertex reference. Furthermore, similar group differences were observed for both inverted faces and inverted houses, suggesting a lack of face specificity for the attenuation of the N170 inversion effect in ASD.

**Conclusion:**

The present findings refine understanding of face processing ERPs in ASD. These data provide only modest evidence for highly-selective ASD-sensitive ERP features, and underscore the sensitivity of these biomarkers to ERP reference scheme. These schemes have varied across published studies and must be accounted for in future studies of the relationship between these commonly acquired ERP characteristics, genotype, and ASD.

**Electronic supplementary material:**

The online version of this article (10.1186/s13229-018-0220-x) contains supplementary material, which is available to authorized users.

## Background

Autism spectrum disorders (ASDs) represent a continuum of neurodevelopmental impairments characterized by deficits in social interaction, communication, and restricted interests, or repetitive behaviors. ASDs are highly heritable and commonly polygenic in origin [1, 2]. The complex nature of the phenotype complicates its association with specific genetic factors. A focus on more specific biobehavioral or neurophysiological characteristics mediating genetic influences on ASD (intermediate phenotypes, or endophenotypes) carries the potential to facilitate gene discovery and to elucidate the neurocognitive pathways by which genes influence complex social behavior [3]. To be considered an endophenotype, a trait should reliably differentiate ASD individuals from the general population, be heritable, quantitative, and observed not only in individuals diagnosed with ASD, but also in their unaffected family members at a higher rate than in the general population [4, 5].

From early infancy, children with ASD show atypicalities in social communication, such as lack of human face preference over objects, and neurophysiological indices of face processing have been suggested as a potential ASD endophenotype [6–9]. In childhood, individuals with ASD perform poorly in facial emotion recognition across multiple expressions [10], face recognition, and discrimination [11–14]; therefore, such children may employ different neurophysiological mechanisms for face processing than typically developing controls [15, 16]. Early stages of face processing in ASD have been extensively studied using event-related brain potentials (ERPs). This methodology provides direct measurement of neuronal activity with millisecond time resolution and thus permits the detection of the timing and magnitude of neural responses corresponding to distinct stages of cognitive processing. The processing of facial stimuli is reflected by the prominent ERP components P1 and N170, peaking within the first 200 ms after a stimulus onset [17–23]. Multiple studies have reported abnormalities in these components in ASD populations [16, 24–31]; however, a systematic review pointed to discrepancies in the results [32]). A recent meta-analysis of N170 characteristics in ASD indicated that only delay in N170 latency consistently differentiated ASD from controls; however, even this effect was of a small size [33]. Here, we performed a more focused analysis of data from published studies narrowed on theoretical grounds to include only those related to face versus object superiority and face inversion effects.

Face over object superiority refers to the fact that, in the general population, N170 latency is prolonged in response to objects compared to faces [34, 35]. This possibly reflects network optimization of coding face stimuli in humans. The face inversion effect manifests behaviorally as more accurate performance on both memory and perceptual tasks when faces are oriented upright than when inverted (i.e., upside down). In the general population, this inversion effect is substantially larger for faces than non-face objects [36, 37]. Reduced face inversion effects on performance [11, 38, 39] have been observed in the ASD population and have been interpreted as evidence of the abnormal functioning of the face-specific system and/or application of part-based processing strategies [40] instead of the holistic approaches that characterize typical face perception [36, 41, 42]. Indeed, ASD individuals favor local/part-based processing over configurational processing [43]. Both P1 and N170 components of ERPs have been shown to index the face inversion effect in the general population [17, 20, 44].

The heritability of behavioral measures of face preference has been supported in recent twin research [45]. It has also been suggested that relatives of ASD probands have impaired face recognition and atypical patterns of face processing, as observed in ASD-affected subjects [46–49]. Moreover, studies of unaffected twins [6, 50] have demonstrated heritability of ERPs elicited by faces, including both neutral and emotional expressions. Familial aggregation of face-sensitive ERP characteristics has been observed among the parents of ASD probands [46]. Our study examined familial aggregation of an array of ERP characteristics related to face processing which have been previously implicated in ASD. Consistent with available reports [23, 25, 27, 29], we hypothesized that these ERP characteristics would be observed in our ASD subjects, and that unaffected first degree relatives of individuals with ASD would exhibit attenuated versions of these effects. This would provide data consistent with a general hypothesis that face-related ERPs reflect genetically transmitted risk for ASD.

Another aim was to clarify the effect of the electroencephalography (EEG) reference type on the hypothesized group differences. Historically, ERP studies of ASD have employed different reference schemes, and this may have contributed to discrepancies in their findings. The choice of reference electrode is known to have substantial effects on local EEG [51–53] and particularly on face-related ERPs [54]. There is currently no universally accepted “gold standard” and the selection of reference scheme for studies of face-related ERPs in ASD has been highly inconsistent. We undertook a systematic re-appraisal of published results, taking into account this often-overlooked confound. The use of multiple reference schemes in the original data collection described in this report enabled the comparison of our results with previous studies that have employed distinct reference schemes for quantifying various ERP associations with ASD.

## Methods

### Reappraisal of ERP abnormalities in ASD based on previously published studies

From 23 studies of the N170 component in ASD identified by a recent systematic review [32], nine [16, 24–31] included a comparison of the ERP response to upright face stimuli with responses to either inverted face stimuli or non-face objects. Of these nine studies, eight [16, 24–30] assessed the face over object superiority effect and five [16, 27–29, 31] assessed the face inversion effect (Table 1). Extending the previous review [32], we calculated weighted effect size of between-group differences assessed from the published data. The GPower program [55] was used to estimate the minimum group size needed to detect the effects of interest with at least 80% power and alpha of 0.05.

**Table 1:** Summary of studies involving face perception ERP’s in non-intellectually disabled ASD subjects with effect size estimates (Cohen’s d and unbiased Hedges g)

Study	Demographics: final N (age range: mean ± SD), males %)	EEG system/Reference	Stimuli	Task	N170 latency face superiority (effect size d/g*)	N170 amplitude face inversion (effect size d/g*)	P1 amplitude face inversion (effect size d/g*)	Other ERP findings
Tye et al., 2013 [31]	ASD: 19 (8–13:11.7 ± 1.7, 100%) ASD + ADHD: 26 (8–13:10.6 ± 1.7, 100%) ADHD: 18 (8–13:10.5 ± 1.9, 100%) TD: 26 (8–13:10.6 ± 1.8, 100%)	62 Acticap/Average	Female faces upright/inverted with gaze direct/averted **No fixation cross**	Count flags among fixation		ASD/ASD + ADHD vs. TD + ADHD: d = 0.38, n.s.	n.s.	Enhanced N170 amplitude in Left Hemisphere
Churches et al., 2012 [30]	ASD: 10 (30 ± 6.2, 100%) TD: 13 (30 ± 4.8, 100%)	**32 Neuroscan/Nose**	Faces/face-like objects/non-face stimuli **No fixation cross**	Motor response on flower	n.s.			Smaller N170 amplitude for non-face like objects
Webb et al., 2012 [29]	ASD: 32 (18–44:23.1 ± 6.9, 94%) TD: 32 (18–43:23.7 ± 6.7, 91%)	128 EGI/Average	Faces/houses, upright/inverted, scrambles faces	Motor response on scrambled faces	0.56/0.54	n.s.	+, *p* < 0.05	Face inversion effect on P1/N170 slope: d = 0.63/g* = 0.61
McPartland et al., 2011 [27]	ASD: 36 (11.2 ± 3.4, 89%) TD: 18 (12.6 ± 2.4, 83%)	256 EGI/Average	Faces/houses, upright/inverted (inverted houses not analyzed)	Motor response on repeated stimuli	0.63/0.61	0.41/0.40	−0.10/−0.10, n.s.	
*Hileman* et al.*, 2011 [28]*	*ASD: 27 (9.4–17.4:13.2 ± 2.7, 85%)* *TD: 22 (9.0–16.9:14.3 ± 2.0, 82%)*	*128 EGI/Average*	*Emotional faces, upright/inverted vehicle* **No fixation cross**	*Count female faces/left pointing cars*	*−0.65/− 0.63, n.s.*	*−0.43/− 0.42, n.s.*	*1.09/1.05*	*Strange data: positive N170, no face inversion effect even in TD*
Churches et al., 2010 [26]	ASD: 12 (31.4 ± 6.7, 100%) TD: 13 (29.3 ± 4.6, 100%)	**32 Neuroscan/Nose**	Faces/Chairs **No fixation cross**	Stimulus repetition detection task	n.s.			Modulation by attention
O’Connor et al., 2007 [25]	ASD: 15 (18–41:23 ± 4, 100%) TD: 15 (19–37:18 ± 15,100%)	128 EGI/Average	Sad/neutral faces/eyes/months/objects	Motor response on sad	0.67/0.63			
Webb et al., 2006 [24]	ASD: 27 (2.7–4.5:3.7 ± 0.3) TD: 18 (2.7–4.5:3.7 ± 0.6) DD: 18 (2.7–4.5:3.7 ± 0.4) % of males not reported	64 EGI/Average	Familiar and unfamiliar faces and objects **No fixation cross**	No task	0.56/0.54			N170 precursor Larger ERPs to objects
McPartland et al., 2004 [16]	ASD: 9 (15–42:21 ± 8, 89%) TD: 15 (16–37:24 ± 6, 93%)	128 EGI/Average	Faces/furniture, upright/inverted, butterflies as targets (inverted furniture not analyzed) **No fixation cross**	Count butterflies	1.19/1.10	0.18/0.17, n.s.		
Dawson et al., 2005 [45]	Parents of ASD: 21 (29–52:38.5, 48%) Parents of TD: 21 (28–51:38.9, 38%)	128 EGI/Average	Faces, inverted/upright, chairs **No fixation cross**	Count scrambled faces	0.62/0.59			Smaller N170 amplitude in Right Hemisphere for faces

Note: One entry (Hileman et al., 2011) is italized in the table due to highly atypical results. The text in bold highlights the studies’ characteristics that might have influenced the results, e.g. suboptimal reference schemas, absence of fixation cross or inclusion of female participants

### New data collection

#### Subjects

Our study sample consisted of 59 autistic spectrum disorder (ASD), 40 unaffected siblings (US), and 56 unrelated Control (UC) males aged 12–21 and 18 unaffected fathers (UF) of families with more than one child with ASD (multiplex families) aged 30–56. All subjects in this data collection were male on the basis of study design (a longitudinal study of children with autism spectrum disorder and their male siblings, US NIH HD 042541). Exclusionary criteria for participation were a history of brain trauma or seizures and/or severe hearing/visual/physical disabilities. All ASD probands were verbal and were characterized according to (1) the Autism Diagnostic Interview–Revised (ADI–R) [56]; (2) the Social Responsiveness Scale (SRS) [57]. The latter was obtained on all subjects in the study including UC subjects, as a measure of quantitative variation in autistic social impairment, ranging from subtle, subclinical autistic-like traits to clinical-level symptomatology; (3) expert clinician diagnosis with final research diagnostic determination according to Diagnostic and Statistical Manual of Mental Disorder-IV (DSM-IV), derived from the information gathered in 1–3. The use of ADI-R and expert clinician assessment/diagnosis reasonably ensures that the probands in this study were affected by ASD as suggested by a previous study showing that ascertainment by ADI-R and historic clinical diagnosis alone results in research diagnosis using ADI-R and The Autism Diagnostic Observation Schedule (ADOS) [58] 98% of the time [59]. For the purposes of this study, we define “verbal” as operationalized by ADI-R item 30 (overall level of language), endorsing “functional use of spontaneous, echoed, or stereotyped language that, on a daily basis, involves phrases of three words or more that at least sometimes included a verb and are comprehensible to other people.”

All non-ASD subjects were recruited from the community or from a group of siblings of non-ASD child psychiatric patients enrolled in the same longitudinal study at Washington University; they underwent clinical diagnostic screening to confirm non-ASD status if their SRS score was greater than 60 T [57]. All subjects were native English speakers. After the exclusion of subjects with random behavioral performance, poor ERP signal (see the “EEG Recording and Analysis” section), our analysis sample consisted of 49 ASD subjects (seven meeting DSM-IV diagnostic criteria for autistic disorder (299.0) and 42 meeting DSM-IV diagnostic criteria for Asperger’s disorder or pervasive developmental disorder, not otherwise specified (PDD-NOS) (299.80)), 36 US, 53 UC, and 18 UF subjects (see Table 2 for the sample details). Four US and 10 UC subjects had community diagnoses of attention deficit hyperactivity disorder (ADHD). The total number of *families* represented by the ASD, US, and UF subjects was 126. Mean (± SD) full-scale intelligence quotient (IQ) for the ASD subjects was 106 ± 31; three ASD subjects had full-scale IQ < 70; verbal IQ ranged from 48 to 152, with a mean of 103 and a standard deviation of 21. The study was approved by the Washington University School of Medicine Human Research Protection Office. Individual informed consent was obtained from all subjects aged 18 and older and from parents of subjects below age 18. All subjects below age 18 who had capacity to provide assent were afforded opportunity to do so and were only included in the study if they gave assent.

**Table 2:** Sample description

	Autism spectrum disorders, ASD	Unaffected siblings, US	Unaffected controls, UC	Unaffected fathers, UF
*n*	49	36	53	18
Age, years	15.2 ± 2.7	15.5 ± 2.5	15.5 ± 2.0	44.1 ± 7.1
SRS	90 ± 31	23 ± 18	21 ± 17	29 ± 14
Caucasian	92%	89%	76%	94%
Right-handed	78%	83%	86%	83%
Multiplex family	35%	6%	0%	100%
Medication free	27%	58%	83%	No data
Task performance	69–100	78–100	75–100	84–100
	95 ± 7	96 ± 6	96 ± 6	98 ± 4

Note: SRS scores were unavailable for eight UF subjects. The family history of ASD was examined and multiplex family status was designated if there was more than one ASD child in the family, in all other case the family was considered simplex (e.g., including families in which the ASD-affected child was the only child in the family)

#### Experimental procedure

The experiment was calibrated to procedures described by Webb et al. 2012 [29] through direct consultation with their research program. Face stimuli, which were kindly provided by Dr. Webb’s group, consisted of gray-scale digital images of faces and houses presented for 300 ms against a gray background on a computer monitor. All facial images were standardized so that the eye region was aligned with the center of the screen, where a fixation cross was presented during the inter-stimulus interval (pseudorandom duration from 1700 to 2000 ms). This was done to help ensure fixation on the eyes, which can be compromised in ASD subjects [14, 60] and contribute to observed hypoactivation of “face-specific” systems in ASD [61, 62]. Stimuli, subtending a 4.2 × 3.3 degree visual angle for faces and 2.8 × 3.3 for houses were presented in four pseudorandom 58-trial blocks and included five different stimulus categories: upright faces, inverted (upside down) faces, upright houses, inverted houses (*n* = 50 in each category), and scrambled faces (parts of a face image with random placement and orientation, *n* = 32). Subjects were instructed to keep their gaze at the fixation point and press a button when a scrambled face appeared. This secondary task was introduced to ensure that the subjects were attending to the stimuli; it also allowed us to identify “random performers”, i.e., subjects who missed more than 32% target stimuli (corresponding to 50% confidence interval with 0.05 alpha). Five subjects, all from the ASD group, were excluded from further analysis based on this criterion. There were no “random performers” in any of the other groups. After the exclusion, performance accuracy in the ASD group ranged from 69 to 100%, and there was no significant difference among the groups, with Mean ± SD values being 95 ± 7, 96 ± 6, and 96 ± 6 for ASD, US, and UC groups, respectively. In addition, we videotaped the subjects and video recordings of those subjects who missed over 15% of trials were reviewed to confirm that all subjects included in the analysis had maintained eye gaze on the computer display during the task. There were no additional exclusions based on this review.

#### EEG recording and analysis

Synamps-2 bioamplifiers (Compumedics/Neuroscan, El Paso, TX) were used for the EEG recording. Thirty sintered Ag/AgCl electrodes embedded into an elastic Quik Cap (Compumedics/Neuroscan, El Paso, TX) were positioned according to the standard 10–20 montage plus one ground electrode. A nose electrode served as a reference. The montage also included left and right mastoid electrodes that provided a reference for the resting EEG and other ERP paradigms not reported here. The data were re-referenced offline to (1) infinity with the REST technique, which has been suggested to have superior performance over average reference [52, 63]; (2) average reference, which has been most commonly implemented in previous research on face abnormalities in ASD; and (3) the vertex (Cz) reference, as potentially optimal for the detection of face-sensitive brain generators that purportedly manifest themselves as a negativity at parietal sites and a positivity at central sites, also known as vertex positive potential (VPP), - the face-sensitive ERP component described in earlier literature that resembles N170 with respect to its time course and functional properties [54]. Electrode impedances were kept below 5 KΩ. Electrooculography (EOG) electrodes, positioned above and below the left eye (vertical EOG) and laterally to each eye (horizontal EOG), were used for monitoring eye movements. Hardware filters were set at 0.01–100 Hz. The sampling rate was 500 Hz.

The data were bandpass filtered (0.1–30 Hz, finite impulse response (FIR), 48 dB) and then epoched using periods spanning 100 ms pre-stimulus onset to 500 ms post-stimulus onset. The baseline was defined as the mean amplitude in the pre-stimulus interval of 100 ms. Automatic artifact rejection excluded trials in which the signal amplitude exceeded ± 120 μV in the EEG and ± 150 μV in the EOG channels. ERPs were averaged separately within each stimulus category. Four subjects (one ASD, two US, and one UC) had to be excluded from the analysis due to the limited number of trials available for averaging (< 10). After the exclusion, the number of trials ranged from 10 to 50 in individual subjects and did not differ significantly between the groups in any stimulus category, with means of 35, 38, and 38 for ASD, US, and UC, respectively. All individual averaged ERP waveforms were visually inspected. In a small portion of recordings, P1 or N170 peaks could not be identified with confidence at electrodes of interest due to the lack of a single dominant peak within the peak detection window, which could have resulted from low amplitude and overall noisy recording. Since ambiguity in peak detection could potentially lead to inaccurate measurement of the peak latency, a key dependent variable in our analyses, these recordings (five ASD, one US, three UC, 5% of the sample) were excluded from subsequent analyses. In addition, to generate a single measure for each of the contrasts of interest (e.g., upright and inverted faces), we computed “difference waves” by subtracting ERP waveforms elicited by different stimulus categories.

The following ERP components (named according to peak latency and polarity) were analyzed: P1 (also known as P120 or P100) with a maximum at occipital sites (O1, O2) and N170 with a maximum at lateral parietal sites (P7, P8). Average amplitude was measured in a time window of ± 20 ms around the peak, which was determined for each subject separately within the following ranges: 70–170 for P1 and 110–230 ms for N170, as recorded at dominant peak sites (O1/O2 for P1 and P7/P8 for N170). The new measure introduced by Webb et al. 2012 [31], P1/N170 slope, was calculated as difference between P1 and N170 amplitude divided by difference between P1 and N170 latencies measured at P7/P8 sites.

#### Statistical analysis

A mixed-design analysis of variance (ANOVA) including the within-subject factors “stimulus type” (face vs. houses), “orientation” (inverted vs. upright) and “hemisphere” (left vs. right) and the between-subject factor “group” (ASD/US/UC) was performed separately for each dependent variable (component’s amplitudes and latencies). Partial η^2^ was used to estimate effect sizes. The one-tail Student’s *t* test for independent samples (ASD vs UC, ASD vs US, and US vs UC) was used for testing our primary hypotheses and post hoc analyses. Cohen’s *d* was used to estimate the effect size for these comparisons. As UF could not be directly compared with our younger groups due to the large effect of age on the studied ERP components, data from UF were analyzed separately using within-subject ANOVA. To examine the relationship between P1 and N170 characteristics, we used Pearson’s correlation coefficient. All data analyses were performed separately for each of the four different reference schemes, and Bonferroni correction of *p* values was used to safeguard against type I errors. Within-subjects comparisons were tested using paired *t* tests.

To test for the correlation between ERP measures, IQ, and the SRS scores while controlling for possible confounding effects of age, we computed partial correlations with age entered as a covariate. A total of eight tests were performed, resulting from the combination of two ERP characteristics of interest and four reference schemes.

In addition, to facilitate the comparison of the present results with previous studies, we used the replication Bayes factor statistic, recently introduced by Verhagen and Wagemakers [64]. This was motivated by the fact that the absence of a significant effect in the present study does not necessarily imply a statistical difference between the present study and previous studies that reported a significant effect. The approach suggested by these authors allows one to quantify the extent to which the observed data support the skeptic’s or the proponent’s replication hypothesis with the Bayes factor value (BF). A BF value above 3 is thought to indicate moderate to strong support for replication and values below 1/3 are regarded as evidence for non-replication. BF was calculated by comparing the weighed means of the effect and sample size from previous studies with the respective parameters in the current study. The computations were carried out using an R code available on Dr. Verhagen’s website (http://www.josineverhagen.com/?page_id=76#_blank).

Because the sample included three subjects with IQ below 70 in the ASD group and subjects with ADHD diagnosis (*n* = 10 in the UC group and *n* = 3 in the US group), we have repeated all hypothesis-testing analyses excluding these individuals in order to determine whether their inclusion might have impacted our findings. These follow-up analyses provided a more stringent comparison between non-intellectually disabled ASD subjects and typically developing controls.

Finally, to examine whether poor performance of the secondary “control” task could have affected or confounded the results, we computed correlations between accuracy in the secondary task and ERP variables of interest and re-analyzed the data after applying stricter exclusion criterion (accuracy below 90%).

Neuroscan software was used for pre-processing, and data were imported into MATLAB (Mathworks) for re-referencing to infinity and ERP analyses. Statistical analysis was done with SPSS.

## Results

### Reappraisal of ERP abnormalities in ASD based on previously published studies

Published studies included in our analysis are listed in Table 1, along with relevant methodological details and effect sizes for selected ERP characteristics.

A first purported ERP abnormality, the reduction in face over object superiority of N170 timing in ASD subjects compared with controls, has been observed in five studies [16, 24, 25, 27, 29], although three studies have failed to find a significant between-group difference [26, 28, 30]. Two of these studies [26, 30] were excluded from our analysis due to the lack of data required for effect size calculation. Another study with negative findings [28] was excluded due to highly atypical ERP responses [see Additional file 1]. Analyses of the remaining five studies yielded a weighted average effect size of *d* = 0.68, a medium-size effect according to Cohen’s classification [65], which provided a quantitative estimate of between group differences. However, it is important to note that, because analyses were based on data from studies with positive findings only, this estimate is likely to be overestimated. Of note, parents of children with ASD also exhibited reduced face over object superiority effect of N170 timing as compared to parents of typically developing children [46] with an effect size of 0.62.

A second purported ERP abnormality, diminished effect of face inversion, as reflected in either P1 or N170 amplitudes, was not supported by the accumulated literature. P1 amplitude inversion was examined in four studies [27–29, 31] with only two reporting significant group differences [28, 29]. Weighted effect size estimation also did not support the hypothesis that the reduced P1 face inversion effect is a distinguishing characteristic of ASD (Additional file 1). The N170 amplitude inversion effect was examined in five studies [16, 27–29, 31], among which only one reported a significant group difference [27]. The weighted average effect size from those studies (two negative and one positive) is 0.35, corresponding to small effect size. However, a new composite measure of face inversion effect introduced by Webb et al. [29], the P1/N170 slope, showed a better discrimination between ASD vs. TD. This measure combines P1 and N170 components affected by face inversion and, as noted by Webb et al., “takes into consideration the peak-to-peak change in amplitude over the peak-to-peak change in latency” [29]. This slope index differentiated ASD from neurotypical controls with an effect size of 0.63.

Therefore, collective evidence from previous studies suggests that the two ERP measures related to face processing which warrant strongest consideration as potential ASD endophenotypes are (1) the face over object superiority effect on N170 timing and (2) the face inversion on P1/N170 slope. These ERP measures differentiated ASD from neurotypical subjects with medium effect sizes [0.68 and 0.63, as assessed from [16, 24, 25, 27, 29] and [29], respectively). A power analysis revealed that an effect of this size can be detected with at least 80% power with a sample size of 33 subjects per group. Each group in our sample exceeded this threshold, with the exception of UF.

### New data collection

#### Effects of stimulus type and orientation

**Fig. 1:**
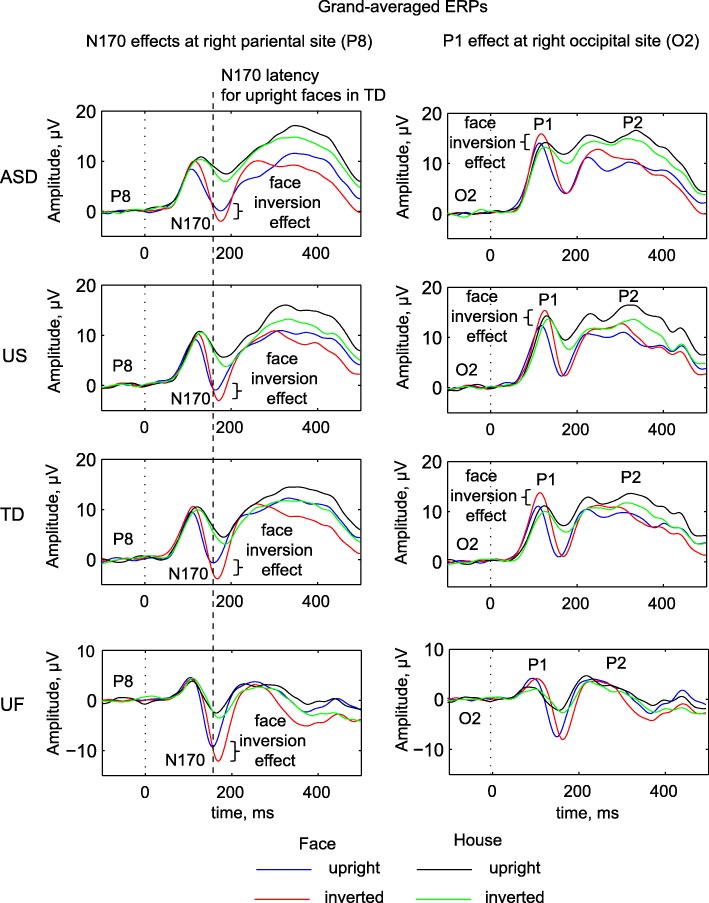
Grand average ERPs, obtained with the vertex reference, in response to upright and inverted faces and houses (coded by different lines) for ASD, US, and UC from right parietal (P8) and occipital sites (O2), to represent N170 and P1 effects. A clear face inversion effect is seen for each group

In our study, the largest amplitudes of P1 and the greatest N170 face inversion effect were observed with the vertex reference. Figure 1 presents grand-averaged ERPs from P8 and O2 electrodes obtained using this reference scheme (scalp topography is shown in Additional file 2). Table 3 summarizes the results of ANOVAs obtained with different reference schemes (statistics are provided in Additional file 3). Consistent with previous studies in non-clinical samples, in our children’s group face stimuli produced earlier and larger N170 component (across all reference schemes employed: main effect of stimulus type on latency was *F*(1, 135) > 35.66, *p* < 0.001, η^2^ > 0.209; main effect of stimulus type on amplitude: *F*(1, 135) > 160.96, *p* < 0.001, η^2^ > 0.544). Similarly, inverted images produced earlier and larger N170 component compared to that produced by upright images (main effect of orientation on latency: *F*(1, 135) > 15.88, *p* < 0.001, η^2^ > 0.105; main effect of orientation on amplitude: *F*(1, 135) > 41.79, *p* < 0.001, η^2^ > 0.236). Although less consistent across reference schemes, similar differences were observed in UF. In addition, in children (ASD, US, and UC) these effects on N170 amplitude showed a hemispheric asymmetry (type X hemisphere interaction: *F*(1, 135) > 9.04, *p* < 0.003, η^2^ > 0.063 hemisphere X orientation interaction: *F*(1, 135) > 18.57, *p* < 0.001, η^2^ > 0.121). Of note, the inversion effect on N170 amplitude was not face-specific in children (no significant interactions involving stimulus type and orientation: all *p* > 0.05, but was larger for faces than houses with average and vertex references in UF (*F*(1, 17) > 7.93, *p* < 0.012, η^2^ > 0.318).

**Table 3:** ANOVA Results

	N170 latency	N170 amplitude	P1 latency	P1 amplitude	P1/N170 slope
General effects					
Type	++++	++++	++++	+++±	++++
	++=±	++++	====	++++	++++
Orientation	++++	++++	++++	++++	++++
	++++	=±++	+++±	====	====
Hemisphere	====	====	++++	====	++++
	====	====	====	====	====
Type X orientation	==±=	====	++++	++++	++++
	++=±	==++	====	====	±±==
Type X hemisphere	+++=	++++	====	==±=	±+++
	====	====	====	====	====
Orientation X hemisphere	==±=	++++	====	±=±+	±=±=
	====	====	====	====	====
Type X orientation X hemisphere	====	====	===+	===±	====
	====	====	=±=+	====	====
Group differences (ASD/US/UC)					
Group	====	====	===±	====	====
Type X group	=±==	====	====	±===	====
Orientation X group	====	===+	====	====	=±==
Hemisphere X group	====	====	====	===±	====
Type X orientation X group	====	====	====	====	====
Type X hemisphere X group	====	====	====	±===	====
Orientation X hemisphere X group	±===	====	====	====	====
Type X orientation X hemisphere X group	====	====	====	====	====

Within each cell, test results are presented respectively for nose, REST, average, and vertex references, in that order. For general effects, upper array is for children; lower array for adults (UF)

Note: ‘+’ codes for significant effects surviving Bonferroni correction, and “±” corresponds to statistical significant for a given singular test, which did not survive Bonferroni correction for multiple comparison (four reference schemes), “=” codes for insignificant effects (*p* ≥ 0.05) for nose/REST/average/vertex references, respectively (e.g., “===+” indicated that the effect is significant only with vertex references). For each factor, the irst line indicates effects for ASD/US/UC combined and the second line is for the separate analysis of UF

Finally, in children, the latency of the earlier P1 component was shorter for upright faces than both houses and inverted faces (type X orientation interaction: *F*(1, 135) > 12.47, *p* < 0.001, η^2^ > 0.085), and P1 amplitude was larger for inverted faces than houses and upright faces (type X orientation interaction: *F*(1, 135) > 36.12, *p* < 0.001, η^2^ > 0.211). In UF, only the main effect of stimulus type was significant for P1 amplitude (*F*(1, 17) > 11.45, *p* < 0.004, η^2^ > 0.402) and the main effect of orientation for P1 latency (F(1, 17) > 7.08, *p* < 0.016, η^2^ > 0.294). The face over object superiority in latency and the face inversion effects for P1 and N170 were not correlated (*p* > 0.05), suggesting distinct underlying mechanisms involved in the modulation of these ERP components. In addition, we confirmed the sensitivity of P1/N170 slope to face inversion [29]. Extending this finding to the child population: P1/N170 slope was steeper for faces than houses (main effect of stimulus type: *F*(1, 135) > 172.99, *p* < 0.001, η^2^ > 0.562) and for inverted rather than upright stimuli (main effect of orientation: *F*(1, 135) > 62.36, *p* < 0.001, η^2^ > 0.316); the inversion effect was larger for faces than houses (type X orientation interaction: *F*(1, 135) > 18.53, *p* < 0.001, η^2^ < 0.121). In UF, only the main effect of stimulus type was significant (*F*(1, 17) > 27.99, *p* < 0.001, η^2^ > 0.622).

#### Group comparisons

Neither N170 nor P1 differentiated ASD/US and UC groups consistently across all reference schemes. There were no significant main effects of group on P1/N170 amplitude and latency or interaction of any studied factors with group (Table 3), except the group X orientation interaction, which survived Bonferroni correction under the vertex reference. Following this significant effect (*F*(1, 135) = 5.14, *p* < 0.01, η^2^ = 0.071), the general N170 amplitude inversion effect was calculated as the difference between inverted and upright stimuli averaged irrespective of stimuli type (face and houses) and hemisphere (P7 and P8 electrodes). This general inversion effect was equal to 0.57 ± 0.38, 1.09 ± 0.39, and 2.17 ± 0.33 in ASD, US, and UC groups, respectively, and post hoc analyses revealed that both ASD and US groups differed significantly from the UC group (*p* ≤ 0.05, Bonferroni uncorrected).

Table 4 summarizes the results of planned *t* test comparisons. We note that these results of group comparison were unchanged when the “difference wave” obtained by subtracting one condition from another was used instead of ERPs for individual conditions. None of the tested ERP effects were significantly correlated with SRS scores in any of the studied groups (rs < 0.2, ps > 0.1; scatterplots are provided in Additional file 4). Below, we provide more detailed results pertaining to specific group differences as hypothesized based on previous literature.

**Table 4:** Tests of study hypotheses (one-sided *t* test, Bonferroni uncorrected) and post hoc follow-up of significant ANOVA effect (two-sided *t* test, Bonferroni uncorrected)

Hypotheses	Reference scheme	Group difference, t/p/d			Main effect, t/p			
		ASD vs. UC	UC vs. US	US vs. ASD	ASD	UC	US	UF
1. Face over objects superiority effect (difference between N170 latency for face and houses upright at P8)	Nose	1.12/.13/.22	.42/.34/.09	1.56/.06/.34	.91/.18	2.52/< .01	3.52/< .01	1.40/.09
	REST	1.43/.08/.28	.72/.23/.16	*1.92/.03/.42*	1.88/.03	4.64/< .01	4.07/< .01	2.28/.02
	Average	**2.50/< .01/.49**	.88/.19/.19	1.53/.06/.34	1.68/.05	7.10/< .01	4.85/< .01	2.86/< .01
	Vertex	1.25/.10/.25	.23/.41/.05	1.05/.15/.23	1.93/.03	4.03/< .01	4.6/< .01	2.03/.03
2. Face inversion effect on P1/N170 slope (difference between face upright and face inverted at P8)	Nose	.84/.20/.17	.39/.34/.08	.48/.31/.10	4.17/< .01	5.10/< .01	5.99/< .01	.99/.17
	REST	*1.95/.02/.39*	1.22/.12/.26	.68/.25/.10	3.72/< .01	6.07/< .01	4.59/< .01	1.14/.14
	Average	1.32/.09/.26	1.44/.08/.31	.06/.47/.01	3.02/< .01	6.92/< .01	4.03/< .01	.53/.30
	Vertex	1.46/.07/.29	1.28/.10/.28	.24/.41/.05	3.03/< .01	6.58/< .01	3.89/< .01	.72/.24
3. Post hoc follow-up*: Inversion effect on N170 amplitude (difference between upright and inverted stimuli—face and houses—at P8/P7)	Nose	.19/.85/.04	.30/.77/.06	.09/.93/.02	2.60/.01	3.62/< .01	2.87/.01	.99/.34
	REST	1.64/.10/.32	.44/.66/.09	1.03/.31/.23	3.25/< .01	4.66/< .01	1.23/.22	.28/.78
	Average	*2.11/.04/.42*	1.79/.08/.39	.24/.81/.05	1.99/.05	3.33/< .01	1.42/.16	.61/.55
	Vertex	**3.11/< .01/.62**	*1.98/.05/.43*	.92/.36/.11	.39/.70	3.79/< .01	1.99/.05	.15/.88

Note: *two-sided *t* test; The results of between group comparisons are italized when the difference is significant, and in bold when the results of comparison survived Bonferroni correction. The t/p/d in the colunm headings corresponds to t-test statistics, *p* value of significance and d Cohen's effect size

### Is the face over object superiority effect reduced in ASD?

The N170 latency was significantly shorter for faces than houses for UC and US, but not ASD children, irrespective of reference type (Table 4). In the UF group, the difference did reach significance but only with the average reference scheme. In spite of a qualitative difference, the magnitude of the face superiority effect (difference between N170 latencies in response to faces and houses) did not consistently differentiate ASD from other groups. A significantly reduced face superiority effect was observed in ASD subjects as compared to UC only under the average reference. The reduction in the face superiority effect was due to delayed N170 latency for faces in ASD children as compared to UC (Additional file 5). The difference between US and UC did not reach significance for any of the reference schemes.

#### Comparison with previous studies

Results of the Bayesian analysis. In regard to ASD vs. UC difference, our results obtained for the average reference data (effect size of 0.55) provided strong support for the previous findings (the weighted effect size of 0.68) as indicated by Bayesian factor of 10.2. However, results obtained under other reference schemes are more consistent with the non-replication hypothesis (0.2 < BF < 0.6). As for the US vs. UC difference, the effect size with average reference was 0.19, which is much smaller than that reported by Dawson and colleagues [46] for parents of ASD children (*d* = 0.63). Bayesian analysis was equivocal for the result in the average reference (BF = 0.4) and consistent with non-replication for the other reference schemes (0.2 < BF < 0.3). In addition, our UF group showed a significant (*p* < 0.01, Table 4) face over object superiority effect of 9.2 ± 13.7 ms: N170 latencies were 152.2 ± 12.5 ms for faces and 161.4 ± 19.5 for houses, respectively. However, this effect appears to be more consistent with the data reported by Dawson and colleagues [46] for control parents (10.5 ± 10.2 ms) than for parents of ASD children (3.6 ± 12.1 ms).

### Is the face inversion effect on P1/N170 slope diminished in ASD?

**Fig. 2:**
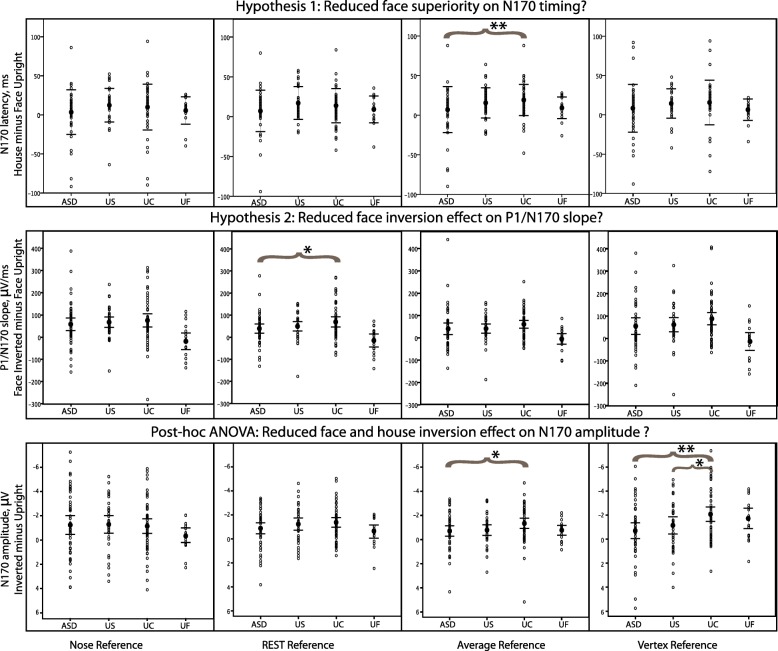
Results of analysis of variance statistics. The figure depicts three ERP phenotypes (three separate lines of panels: 1–3) for four reference schemes (four columns of panels). Individual subjects represented as dots organized by groups: ASD, US, UC, and UF along the *X* axis in each of 12 panels. Brace indicated the significant between group difference (*significant but Bonferroni uncorrected, **significant with Bonferroni correction)

The P1/N170 slope at P8 was significantly steeper for inverted than upright faces, but, contrary to our expectations, this effect showed no significant group differences and was observed in all studied groups of children under all reference schemes irrespective of the diagnosis or family type (Table 4, Fig. 2). Furthermore, the face inversion effect, computed as the difference between peak values obtained in inverted and upright conditions for P1 and N170 amplitudes, also failed to differentiate the study groups (Additional file 5, except N170 amplitude inversion with vertex reference related to general N170 amplitude inversion effect, which is discussed in detail below).

#### Comparison with previous studies

Overall, Bayesian analysis was inconclusive, i.e., provided little evidence either in support of or against the group differences reported previously (0.3 < BF < 2.9, with *d* = 0.29 and BF = 0.7 for average reference).

### Are the results affected by the inclusion of subjects with lower IQ and ADHD symptomatology?

To examine whether the findings might be influenced by the inclusion of individuals with low IQ and/or ADHD diagnosis, the above hypothesis-testing analyses were repeated after the exclusion of four US and 10 UC subjects with ADHD diagnosis and three ASD subjects with full-scale IQ < 70. This exclusion did not significantly impact the pattern of results described above. Moreover, our ERP effects of interest were not correlated with IQ scores (all ps < 0.15). Thus, the results obtained for P1 and N170 in our original analyses are unlikely to be driven by the inclusion of either low IQ or ADHD subjects in the analysis.

### The role of performance in the secondary (control) task

A re-analysis of data after the application of stricter subject exclusion criteria based on the performance in the secondary task (responding to less than 90% of the rare target stimuli) did not affect the main findings. Furthermore, no significant correlations between accuracy in the secondary task and ERP variables of interest were observed (all ps > 0.05). Taken together, these results suggest that variability in the performance on the secondary “control” task did not impact the main findings of this study.

## Discussion

### Effects of stimulus type and orientation on P1 and N170 components

Corroborating previous findings in the general population, the N170 component was significantly larger and peaked earlier for faces than for houses, predominantly at the right posterior sites [17–19]. The face inversion effect on N170 reported in previous studies (e.g., [17–19]) was also well replicated in the present study, although our findings challenged its specificity to faces: N170 amplitude was larger for inverted compared to upright images of both faces and houses. We confirmed the sensitivity of a new measure, proposed by Webb and colleagues [29], the P1/N170 slope, to face inversion and extended this finding to the child population: the inversion effect on P1/N170 slope was larger for faces than for houses. Furthermore, our study supported face-related effects on the P1 component [20–23]: P1 latency was shorter for faces than houses, and inverted faces elicited larger P1 than upright faces and houses. The P1 effects were not specific to the right hemisphere and observed both at the left and right occipital sites. It is important to note that P1 and N170 latency facilitation effects for faces were not correlated, suggesting that the “face processing advantage” begins as early as 120 ms post-stimulus and involves distinct underlying mechanisms at different stages of information processing.

### Limited support for the hypothesized ERP endophenotypes for ASD

Many studies have examined the latency of the N170 component in response to face stimuli, although most of them have not found a significant difference between ASD and control groups (17 out of 23, [32]). One potential explanation for this variability of findings could be that N170 represents more general mechanisms of the neural processing of complex visual patterns that are not fully specific to face stimuli. To address this problem we computed the difference in the latencies of N170 elicited in response to objects and faces. In the general population, N170 latency is shorter in response to faces than objects [33, 34], and this superiority effect on N170 timing differentiated ASD from UC.

Our analyses revealed a substantial impact of EEG reference scheme on the results of comparisons between ASD and UC subjects with respect to the studied ERP components. Analysis of published literature (Table 1, [16, 24–30]) showed that five out of eight studies reported a reduced “face over object superiority” on N170 latency among ASD subjects with a weighted average effect size of 0.68. Our present data supported the reduction of the face superiority effect in ASD group as confirmed by Bayesian analysis but only under the average EEG reference scheme. Noteworthy, all studies that reported this effect previously also used the average reference, while two out of three remaining studies [26, 30] utilized a nose reference. Thus, our findings suggest that N170 latency abnormalities in ASD are sensitive to the reference scheme, and the average reference appears to be optimal for detecting that effect.

A primary aim of the present study was to examine familial aggregation of previously reported face-related ERP abnormalities in male relatives of children with ASD. The difference between US and UC groups was of small effect size even with the optimal reference schemes (*d* = 0.19) providing little support for the difference between first-degree relatives (parents of ASD children) and low-risk controls reported previously [46]. Moreover, contrary to a prior report, our sample of unaffected fathers ascertained exclusively from multiplex families showed a significant face over object superiority effect on N170 timing.

The systematic review by Feuerriegel and colleagues [32] suggested that ERP characteristics in response to specific manipulation of face stimuli, such as face inversion, warrant thorough investigation as potential neurophysiological biomarkers of ASD. The present study addressed this issue in a comprehensive manner and found no evidence that the face inversion effect on studied ERP components reliably differentiated ASD from healthy control groups. In a previous study [29], the P1/N170 slope differentiated ASD and controls with a medium effect size (*d* > 0.5), however the present data collection did not replicate this effect (Table 4); moreover neither P1 nor N170 amplitude (Additional file 5) differentiated ASD and controls in this study.

Thus, despite a clear-cut replication of previously reported, general, within-subject effects of face superiority and inversion, the differences between ASD and controls were entirely limited to N170 latency, exclusively derived from the average reference scheme. None of the proposed ERP markers of ASD met the criteria for an endophenotype; notably US and UC groups did not differ significantly with respect to the face over object superiority effect on N170 latency or the face inversion effect on P1/N170 slope. Furthermore, none of the studied ERP components showed significant correlations with a validated dimensional measure of ASD severity (Social Responsiveness Scale score) in any of the studied groups.

### The N170 amplitude inversion effect is not specific to faces

The inversion effect on N170 amplitude differentiated ASD and UC groups, but only with the vertex reference. Contrary to our initial hypotheses, the effect of inversion on N170 amplitude was not specific to face stimuli or hemisphere. Of note, most prior studies of the inversion effect have failed to include a control condition (non-face object inversion) or, when such a condition was included, the results were not reported [16, 27, 28]. The only ASD study that reported data for an object inversion effect on ERP components indeed found that the N170 amplitude inversion effect was reduced in ASD both for faces and houses ([29], see Table 3 on page 585), although this interesting finding was not featured in the discussion. Additional corroborating evidence for non-specificity of the inversion effect to faces comes from a recent behavioral study [66] which reported better performance for upright than inverted images of both faces and cars. Moreover, these non-specific inversion effects were weak and slow to develop in ASD children as compared to controls. Therefore, we conclude that there is little evidence to support the notion that the diminished face inversion effect on N170 amplitude in ASD subjects reflects deficits specific to face processing, as suggested by previous studies [27, 29].

Further support for the common mechanism underlying processing of both inverted faces and objects is derived from studies using neural adaptation paradigms. These studies have shown that inverted objects (houses and Chinese characters) induce an adaptation effect on the N170 component for inverted faces [67, 68]. Additionally, both competition and adaptation effects on the N170 amplitude for inverted faces were larger in the inverted than in upright face context [68, 69], suggesting that the processing of upright and inverted faces recruits distinct neuronal populations of orientation-sensitive neurons [67, 68]. Intracranial recordings [70] have detected activation of both the face-specific and non-specific areas in the lateral occipital cortex in response to face inversion.

It is possible that preference for a part-based over a holistic processing strategy in ASD [43] generalizes to the perception of well-known prototypical objects such as houses and cars and this is what is captured by the non-specific reductions of N170 amplitude inversion. Yet another possibility relates to hypotheses regarding face inversion effects as a function of expertise [71]. Some studies have suggested that only a particular type of expertise, e.g., second-order relational (configural) characteristics [72], or prototype perceptual learning [73, 74] contribute to the effect. Behavioral studies have identified dog image inversion effects in dog experts ([71] but see [37]) as well as hand-writing inversion effects in hand-writing experts [75]; prosopagnosics with special expertise have reported an inability to identify not only faces but birds (among experienced bird watchers) and cows (among an experienced farmer) [76]. Neurophysiological correlates of the face inversion effect have also been reported to be sensitive to expertise [71, 74, 77]. Computer-generated artificial stimuli (“greebles” [77] and prototype-defined checkerboards [74]) have elicited the N170 amplitude inversion effect after extensive laboratory training. Therefore, N170 inversion may index a perceptual learning experience contributing to face and object recognition. Noteworthy, deficits in early experience-dependent learning were recently suggested to underlie the selective impairments in orientation sensitivity along the vertical axis found in ASD children [78].

### Potential moderating and confounding factors

ERP measurements can be affected by a number of factors related to the sample composition (i.e., age, gender, comorbid psychiatric conditions, intellectual variation, and medications), subjects’ understanding of and compliance with the task instruction, and data analysis such as the choice of EEG electrode reference scheme. In the present study, we conducted a series of additional analyses in order to systematically examine the role of these potentially moderating or confounding factors. Details regarding the results of these analyses are elaborated in a corresponding section of Additional files (Additional file 6).

We wish to emphasize here the significant effects of the reference scheme on contrasts between ASD and UC subjects for the studied ERP components. Although within-subject effects of stimulus type and orientation were significant across multiple reference schemes, group differences in P1 and N170 were small and highly dependent on the choice of reference (Tables 3 and 4, Fig. 2). This suggests that to the extent that true differences exist, they may be highly specific to the brain regions uniquely represented by selection of electrodes in which the differences are detected.

### Limitations

Although one of the largest ERP studies of ASD subjects to date, our sample size limited statistical power to detect group effects smaller in magnitude than those reported as positive findings in previous studies. Our study did not include age-matched controls for the fathers of ASD probands (UF), rendering the evaluation of potential ERP abnormalities in this group unfeasible. A direct statistical comparison of UF with other study groups would be inappropriate due to significant age-related ERP differences. However, this group represents a very unique sample of fathers of ASD probands from multiplex families and these data are included in the manuscript for the sake of reporting the entire data set collected in this project. Another limitation is the relatively sparse electrode montage used in the present study (30 EEG electrodes). Although the ERP components of interest (P1 and N170) show a relatively smooth distribution over the respective scalp areas and can be easily identified at several electrodes, a high-density montage would facilitate the detection of peaks in individuals with unusually low ERP amplitude and increase the overall accuracy of amplitude and latency measurements. An additional limitation is the lack of IQ assessments for the unaffected groups, which precluded precise matching of subjects with respect to this variable; inclusion of IQ measurement in future family-based studies will allow for a more rigorous control of potential confounders. We note, however that there is little evidence for a relationship between face ERPs and IQ, and no correlations between the studied ERPs components explored here and IQ measures obtained among the individuals affected by ASD were observed in this study affected by ASD. Finally, although clinician diagnosis with ADI–R confirmation exhibits very strong agreement with categorical designation on the Autism Diagnostic Observation Schedule [58], it was a limitation of the study that data from the latter were not available. The ADOS represents an additional diagnostic standard in ASD research that affords opportunity to test quantitative associations of biomarkers with *autistic severity* among ASD-affected individuals, as measured not only by caregiver report—as was done in this study using the Social Responsiveness Scale—but also by clinician rating.

## Conclusions

In the context of unequivocal replication of (a) the effects of face inversion and (b) face over object superiority on P1 and N170 ERP components (previously reported in the general population), our study did *not* reveal strong evidence for contrasts in these effects between ASD and controls. In our study, the ASD group exhibited the attenuation of face over object superiority on N170 timing in the average reference scheme only, while the reduction of inversion effect on N170 amplitude in this group was significant in the vertex reference scheme only. Moreover, the latter effect was not specific to face and was also observed for houses.

This study was designed to explore whether face-related ERP components reflect the impact of the clinical condition of ASD itself or inherited/background genetic liability, as would be characteristic of an endophenotype. We found no evidence for the aggregation of this face-related ERP variation in first degree relatives, thus suggesting that those features which did relate to ASD were characteristic of the condition itself. The only parameter similarly reduced both in ASD and in unaffected siblings (as compared to neurotypical controls) was the N170 inversion effect; however, this was restricted to a particular reference scheme (the vertex reference) and not specific to face stimuli. These findings have important implications for ongoing studies exploring candidate biomarkers in autism.

Thus, hypothesized group differences in this ERP study whose statistical power compared favorably with the largest ERP ASD studies to date (a) showed either negative or reduced effect sizes for ERPs reported to be associated with ASD in previous studies; and (b) strongly depended on electrode reference scheme, suggesting lack of robust effects. We note that recently, the National Institute of Mental Health launched a major effort in the exploration of electrophysiologic biomarkers for ASD (U19 MH108206, the Autism Biomarkers Consortium for Clinical Trials), for which we urge special attention to the nuances of micro-regional specificity suggested by these findings, noting that these have not been systematically attended to in prior published research in this field.

## Additional files

Additional file 1: Contains tables with mean(SD) values, which was used to calculate weighted effect size in our analysis of previous literature. Data from our study also provided for comparison. (DOC 58 kb)

Additional file 2: Contains figures representing scalp topography of the differences between upright and inverted faces with respect to P1 and N170 amplitudes in the four studied groups (ASD, US, UC, and UF). Note the topography of face inversion effect is different for P1 and N170 amplitude. The P1 face inversion effect shows a clear occipital distribution in ASD, US, and UC groups but is nearly absent in UF. In contrast, the N170 face inversion effect is greater in UF compared to younger groups, and only the younger groups show clear right lateralization of the effect. The topography of the ERP component is similar across reference schemes. (PDF 2037 kb)

Additional file 3: Contains all statistical values for ANOVA analysis. (DOC 32 kb)

Additional file 4: Contains scatterplots depicting the (lack of) relationship between autistic trait severity measured by the Social Responsiveness Scale (SRS, *X* axis) and ERP contrasts of interest obtained with vertex (Cz) reference (*Y* axis): N170 latency for upright face stimuli (A), face superiority effect on N170 latency (B), face inversion effect on N170 amplitude (C), and P1 amplitude (D). Each dot represents an individual subject. Group membership is coded by color: red filled circles indicate children with autistic disorder (299.0), empty red circles stand for PDD_NOS/Asperger (299.80), green empty circles denote unaffected siblings (US), and blue empty circles with unrelated controls (UC). In general, these figures illustrate the lack of significant correlations between the ERP effects of interest and SRS scores in any of the studied groups. (PDF 503 kb)

Additional file 5: Contains supplementary analysis performed for checking additional ERPs characteristics, underlying the main study hypotheses: N170 latency for faces, P1, and N170 amplitude inversion effects. Significant ASD vs TD difference in N170 latency for faces underlie reduced face superiority effect seen for ASD children, presented in Table 4 in the manuscript. The group differences for N170 amplitude inversion effect corresponds with those seen for general N170 inversion effect, represented in Table 4 of the manuscript. No significant group differences were observed for P1 amplitude inversion effect. (DOCX 200 kb)

Additional file 6: Contains discussion of potential moderating and confounding factors that may contribute to the observed discrepancy between results of our and some previous studies. Among considered factors are age, gender, low IQ, and ADHD subjects in our ASD group, attention to stimuli, and reference schemes. (DOC 36 kb)

## Abbreviations

ADHD: Attention deficit hyperactivity disorder
ADI–R: Autism diagnostic interview–revised
ASD: Autistic spectrum disorder
DSM-IV: Diagnostic and Statistical Manual of Mental Disorders-VI
EEG: Electroencephalography
EOG: Electrooculography
ERP: Event-related potential
FMRI: Functional magnetic resonance imaging
IQ: Intelligence quotient
PPD-NOS: Pervasive developmental disorder, not otherwise specified
SRS: Social responsiveness scale
UC: Unrelated controls
UF: Unaffected fathers
US: Unaffected siblings
