# Supplementary material for: Event-related potential (ERP) correlates of face processing in verbal children with autism spectrum disorders (ASD) and their first-degree relatives: a family study
Source: Mol Autism. 2018 Jul 5;9:41. doi: 10.1186/s13229-018-0220-x (PMC6034210; doi:10.1186/s13229-018-0220-x)
Supplement: Supplementary file 1 — Contains tables with mean(SD) values, which was used to calculate weighted effect size in our analysis of previous literature. Data from our study also provided for comparison. (DOC 58 kb) [file 13229_2018_220_MOESM1_ESM.doc]

Additional file 1. Supplementary Table with Mean(SD) values, used to calculate weighted effect size in our meta-analysis. Average Reference schemes were used in all studies with available data.

|  | Face over object superiority effect on N170 latency, P8 electrode | | | | | | | | | | Cohen’s d | N |
| --- | --- | --- | --- | --- | --- | --- | --- | --- | --- | --- | --- | --- |
|  | ASD | | | | | UC | | | | |  |  |
|  | Face Upright | Object Upright | | Difference | | Face Upright | | Object Upright | | Difference |  |  |
| Churches  et al., 2012 | No data | | | | | | | | | | No data | 23 |
| Webb et al., 2012 | ANOVA Stimulus Type*Orientation*Region*Group interaction : F(1,51) = 4.9, p < 0.05 | | | | | | | | | | 0.56 | 64 |
| McPartland et al., 2011 | 201.78(27.2) | 219.38(27.8) | 17.6 | | 181.38(31.6) | | 217(36.4) | | 36.12 | | 0.63 | 49 |
| Hileman et al., 2011* | 176.22(3.74) | 204.88(3.20) | 28.66 | | 166.05(4.15) | | 192.14(3.56) | | 26.09 | | -0.65 | 49 |
| Churches et al., 2010 | No data | | | | | | | | | | No data | 25 |
| O'Connor  et al., 2007 | 192.8(18.5) | 196.5(17) | 3.7 | | 180.3(8.4) | | 193.6(12.8) | | 13.3 | | 0.67 | 30 |
| Webb et al., 2006 | 289(28) | 285(30) | -4 | | 279(26) | | 290(35) | | 11 | | 0.55 | 45 |
| McPartland  et al., 2004 | 200.67(20.4) | 186.44(20.7) | -14.23 | | 180.57(13.4) | | 186.86(16.5) | | 6.26 | | 1.19 | 23 |
| Weighted effect | | | | | | | | | | | 0.66 | 211 |
| Our study | 168.5 (24.3) | 175.5(30.8) | 7 | | 158.3(19.8) | | 177.5(22.0) | | 19.2 | | 0.55 | 102 |
|  | First-degree relatives of ASD proband | | | | UC | | | | | |  |  |
| Dawson et al., 2005* | No data | | 3.6(12.1) | | No data | | | | 10.54(10.2) | | 0.62 | 42 |
| Our study, children | 165.1(20.8) | 180.6(23.3) | 15.5 | | 158.3(19.8) | | 177.5(22.0) | | 19.2 | | 0.18 | 89 |
| Our study,  adults | 152.2(12.5) | 161.4(19.5) | 9.2 | | No age-matched controls | | | | | |  | 18 |
|  | Face inversion effect on N170 amplitude, P8 electrode | | | | | | | | | |  |  |
|  | ASD | | | | TD | | | | | |  |  |
|  | Face Upright | Face Inverted | Difference | | Face Upright | | Face Inverted | | Difference | |  |  |
| Tye et al., 2013 | ANOVA Group*Orientation: F(1,83) = 3.38, p = .07 | | | | | | | | | | 0.38 | 92 |
| Webb et al., 2012 | No data | | | | | | | | | |  |  |
| McPartland et al., 2011 | 0.76(3.6) | 1.52(3.5) | 0.76 | | -0.16(2.0) | | -0.58(2.8) | | -0.42 | | 0.40 | 49 |
| Hileman et al., 2011* | 2.44(0.77) | 3.86(0.84) | 1.42 | | 3.63(0.85) | | 5.4(0.93) | | 1.77 | | -0.43 | 49 |
| McPartland  et al., 2004 | -2.41(3.3) | -3.35(4.7) | -0.94 | | -3.24(3) | | -4.76(3.5) | | -1.52 | | 0.18 | 23 |
|  | Weighted effect | | | | | | | | | | 0.36 | 164 |
| Our Study | -1.94(4.6) | -3.09(4.6) | -1.15 | | -1.38(5.4) | | -3.81(4.5) | | -2.43 | | 0.25 | 102 |
|  | Face inversion effect on P1 amplitude, occipital electrodes | | | | | | | | | |  |  |
| Tye et al., 2013 | No data | | | | | | | | | |  |  |
| Webb et al., 2012 | ANOVA Group*Orientation interaction not reported, but only UC have significant  ANOVA Orientation effect | | | | | | | | | |  |  |
| McPartland et al., 2011** | 9.52(6.5) | 10.40(6.7) | 0.88 | | 7.28(5.0) | | 7.60(5.5) | | 0.32 | | -0.10 | 49 |
| Hileman et al., 2011* | 15.07(1.27) | 15.55(1.23) | 0.48 | | 16.16(1.41) | | 18.10(1.36) | | 1.94 | | 1.09 | 49 |
| Weighted effect | | | | | | | | | | | -0.10 | 49 |
| Our study | 11.87(7.92) | 13.67(8.04) | 1.80 | | 9.45(7.78) | | 11.53(7.50) | | 2.08 | | 0.04 | 102 |

Note:

* data from the right hemisphere was unavailable - the average over hemisphere data were used instead

** data from right and left occipital cortex were averaged

The weighted effect size was calculated as the product of the effect size and the number of subjects in a particular study summed across studies and divided by the total number of subjects in all assessed studies:

Weighted Cohen’s d = ∑(n_i_ * d_i_)/∑n_i_ , where n_i_ and d_i_ are the number of subjects and effect size in a particular study.

The effect size for each particular study was estimated as:

Cohen’s d_i_ = (M_ASD_ – M_UC_)/ √[( (SD_ASD_) ^2^+ (SD_UC_)^2^) / 2], where M_ASD_, SD_ASD_ and M_UC_, SD_UC_ are the Means (M) and Standard Deviation (SD) values reported in those studies for ASD and UC groups, respectively.

In each analysis, the M value represented the effect of interest: the mean difference in N170 latency between face and object stimuli or the mean difference in N170 amplitude between upright and inverted faces. If no SD values for the difference measures were reported, we used SD for upright faces as recommended for the dependent measures design (Morris, 2008). When Mean and SD values were not reported, d was calculated from the F-value as:

d_i_ = √F((n_ASD_+n_uc_)/n_ASD_*n_uc_)*(n_ASD_+n_uc_)/ (n_ASD_+n_uc_-2)), where n_ASD_ and n_uc_ is the number of subjects in ASD and UC groups, respectively (Thalheimer & Cook, 2002).

Two studies [Churches et al., 2012; 2010;] reported neither Means/SD, nor F-statistics, preventing us from including them in the weighted effect size estimation. The results of one study [Hileman et al., 2011] were highly atypical (italic in the table): 1) the N170 component was positive, rather than negative, 2) the face inversion effect on N170 amplitude was in the opposite direction relative to previous studies, including UC [e.g. Bentin et al., 2006; Itier et al., 2004; Taylor et al., 2004; Linkenkaer-Hansen et al.,1998], 3) the reported SD values were approximately 4 times smaller than those reported in the studies of similar size. Therefore, the data from this study were excluded from weighted effect size calculation.

References

Churches O, Baron-Cohen S, Ring H. The psychophysiology of narrower face processing in autism spectrum conditions. Neuroreport. 2012;23(6):395-9. doi:10.1097/WNR.0b013e3283525bc8.

Webb SJ, Merkle K, Murias M, Richards T, Aylward E, Dawson G. ERP responses differentiate inverted but not upright face processing in adults with ASD. Soc Cogn Affect Neurosci. 2012;7(5):578-87. doi:nsp002 [pii] 10.1093/scan/nsp002.

McPartland JC, Wu J, Bailey CA, Mayes LC, Schultz RT, Klin A. Atypical neural specialization for social percepts in autism spectrum disorder. Soc Neurosci. 2011;6(5-6):436-51. doi:10.1080/17470919.2011.586880.

Hileman CM, Henderson H, Mundy P, Newell L, Jaime M. Developmental and individual differences on the P1 and N170 ERP components in children with and without autism. Dev Neuropsychol. 2011;36(2):214-36. doi:933895969 [pii] 10.1080/87565641.2010.549870.

Churches O, Wheelwright S, Baron-Cohen S, Ring H. The N170 is not modulated by attention in autism spectrum conditions. Neuroreport. 2010;21(6):399-403.

O'Connor K, Hamm JP, Kirk IJ. Neurophysiological responses to face, facial regions and objects in adults with Asperger's syndrome: an ERP investigation. Int J Psychophysiol. 2007;63(3):283-93. doi:S0167-8760(06)00320-5 [pii] 10.1016/j.ijpsycho.2006.12.001.

Webb SJ, Dawson G, Bernier R, Panagiotides H. ERP Evidence of Atypical Face Processing in Young Children with Autism. J. Autism Dev. Disord. 2006;36:881–90.

McPartland J, Dawson G, Webb SJ, Panagiotides H, Carver LJ. Event-related brain potentials reveal anomalies in temporal processing of faces in autism spectrum disorder. J Child Psychol Psychiatry. 2004;45(7):1235-45. doi:10.1111/j.1469-7610.2004.00318.x JCPP318 [pii].

Dawson G, Webb SJ, Wijsman E, Schellenberg G, Estes A, Munson J, Faja S. Neurocognitive and electrophysiological evidence of altered face processing in parents of children with autism: implications for a model of abnormal development of social brain circuitry in autism. Dev Psychopathol. 2005;17(3):679-97. doi:S0954579405050327 [pii] 10.1017/S095457940505032

Tye C, Mercure E, Ashwood KL, Azadi B, Asherson P, Johnson MH, Bolton P, McLoughlin G,. Neurophysiological responses to faces and gaze direction differentiate children with ASD, ADHD and ASD+ADHD. Dev Cogn Neurosci. 2013;5:71-85. doi:S1878-9293(13)00002-9 [pii] 10.1016/j.dcn.2013.01.001.

Bentin S, Allison T, Puce A, Perez E, McCarthy G. Electrophysiological Studies of Face Perception in Humans. J Cogn Neurosci. 1996;8(6):551-65. doi:10.1162/jocn.1996.8.6.551.

Itier RJ, Taylor MJ. Face recognition memory and configural processing: a developmental ERP study using upright, inverted, and contrast-reversed faces. J Cogn Neurosci. 2004;16(3):487-502. doi:10.1162/089892904322926818.

Taylor MJ, Batty M, Itier RJ. The faces of development: a review of early face processing over childhood. J Cogn Neurosci. 2004;16(8):1426-42. doi:10.1162/0898929042304732.

Linkenkaer-Hansen K, Palva JM, Sams M, Hietanen JK, Aronen HJ, Ilmoniemi RJ. Face-selective processing in human extrastriate cortex around 120 ms after stimulus onset revealed by magneto- and electroencephalography. Neurosci Lett. 1998;253(3):147-50. doi:S0304394098005862 [pii].

Morris, S. B. Estimating Effect Sizes From Pretest-Posttest-Control Group Designs. Organizational Research Methods, 20008:11(2), 364-386. [doi.org/10.1177/1094428106291059](http://doi.org/10.1177/1094428106291059) .

Thalheimer, W., & Cook, S. (2002, August). How to calculate effect sizes from published research articles: A simplified methodology. Retrieved April 20, 2016 from <http://work-learning.com/effect_sizes.htm>.
