# Supplementary material for: Event-related potential (ERP) correlates of face processing in verbal children with autism spectrum disorders (ASD) and their first-degree relatives: a family study
Source: Mol Autism. 2018 Jul 5;9:41. doi: 10.1186/s13229-018-0220-x (PMC6034210; doi:10.1186/s13229-018-0220-x)
Supplement: Supplementary file 2 — Contains figures representing scalp topography of the differences between upright and inverted faces with respect to P1 and N170 amplitudes in the four studied groups (ASD, US, UC, and UF). Note the topography of face inversion effect is different for P1 and N170 amplitude. The P1 face inversion effect shows a clear occipital distribution in ASD, US, and UC groups but is nearly absent in UF. In contrast, the N170 face inversion effect is greater in UF compared to younger groups, and only the younger groups show clear right lateralization of the effect. The topography of the ERP component is similar across reference schemes. (PDF 2037 kb) [file 13229_2018_220_MOESM2_ESM.pdf]

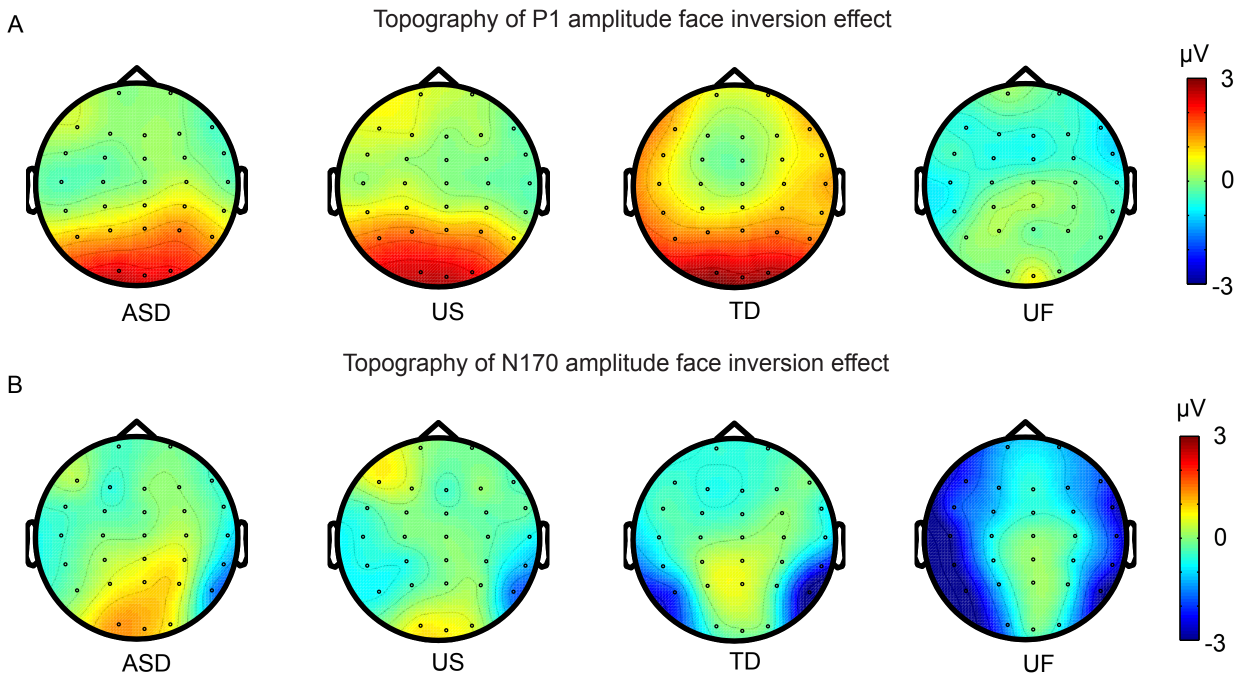
A
Topography of P1 amplitude face inversion effect
ASD
US
TD
UF
μV
3
0
-3
B
Topography of N170 amplitude face inversion effect
ASD
US
TD
UF
μV
3
0
-3
