# Supplementary material for: Event-related potential (ERP) correlates of face processing in verbal children with autism spectrum disorders (ASD) and their first-degree relatives: a family study
Source: Mol Autism. 2018 Jul 5;9:41. doi: 10.1186/s13229-018-0220-x (PMC6034210; doi:10.1186/s13229-018-0220-x)
Supplement: Supplementary file 3 — Contains all statistical values for ANOVA analysis. (DOC 32 kb) [file 13229_2018_220_MOESM3_ESM.doc]

Additional file 3. Supplementary tests of study hypotheses (one-sided t-test, Bonferroni uncorrected).

|  |  | Group difference, t/p/d | | | Main effect, t/p | | | |
| --- | --- | --- | --- | --- | --- | --- | --- | --- |
| Hypotheses |  | ASD vs. UC | UC vs. US | US vs. ASD | ASD | UC | US | UF |
| 1 supplementary: N170 delay for faces  (N170 latency for upright faces at P8) | Nose | .93/.17/.18 | .62/.27./13 | .27/.39/.06 |  |  |  |  |
|  | REST | 1.34/.09/.27 | .29/.38/.06 | .97/.17/.21 |  |  |  |  |
|  | Avr | 2.33/.01/.46 | 1.56/.06/.34 | .67/.25/.15 |  |  |  |  |
|  | Cz | 2.08/.02/.41 | 1.15/.14/.25 | .85/.19/.19 |  |  |  |  |
| 2a supplementary: Face inversion effect on N170 amplitude (difference between face upright and face inverted at P8) | Nose | 1.44/.07/.28 | .78/.22/.17 | .67/.25/.15 | 1.52/.06 | 3.52/<.01 | 2.69/<.01 | .32/.37 |
|  | REST | 2.09/.02/.41 | 1.28/.10/.28 | .61/.27/.13 | 2.35/.01 | 5.63/<.01 | 2.81/<.01 | 1.33/.10 |
|  | Avr | 2.18/.02/.43 | 1.70/.05/.37 | .29/.38/.06 | 2.94/<.01 | 5.57/<.01 | 2.85/<.01 | 2.45/.01 |
|  | Cz | 2.73/<.01/.54 | 1.88/.03/.41 | .52/.30/.11 | 3.29/<.01 | 6.54/<.01 | 3.09/<.01 | 4.13/<.01 |
| 2b supplementary: Face inversion effect on P1 amplitude (difference between face upright and face inverted at averaged over O1 and O2 electrodes) | Nose | .50/.31/.10 | .45/.33/.10 | .98/.16/.21 | 3.87/<.01 | 4.30/<.01 | 5.02/<.01 | .82/.21 |
|  | REST | .97/.17/.19 | .04/.48/.01 | 1.00/.16/.22 | 4.25/<.01 | 5.75/<.01 | 6.20/<.01 | .74/.23 |
|  | Avr | .54/.30/.11 | .16/.44/.04 | .66/.25/.14 | 4.61/<.01 | 5.92/<.01 | 5.87/<.01 | .79/.22 |
|  | Cz | .71/.24 /.14 | .63/.26/.14 | .12/.45/.03 | 4.02/<.01 | 6.30/<.01 | 5.25/<.01 | .05/.46 |
