# Supplementary figures and images for: Event-related potential (ERP) correlates of face processing in verbal children with autism spectrum disorders (ASD) and their first-degree relatives: a family study
Source: Mol Autism. 2018 Jul 5;9:41. doi: 10.1186/s13229-018-0220-x (PMC6034210; doi:10.1186/s13229-018-0220-x)

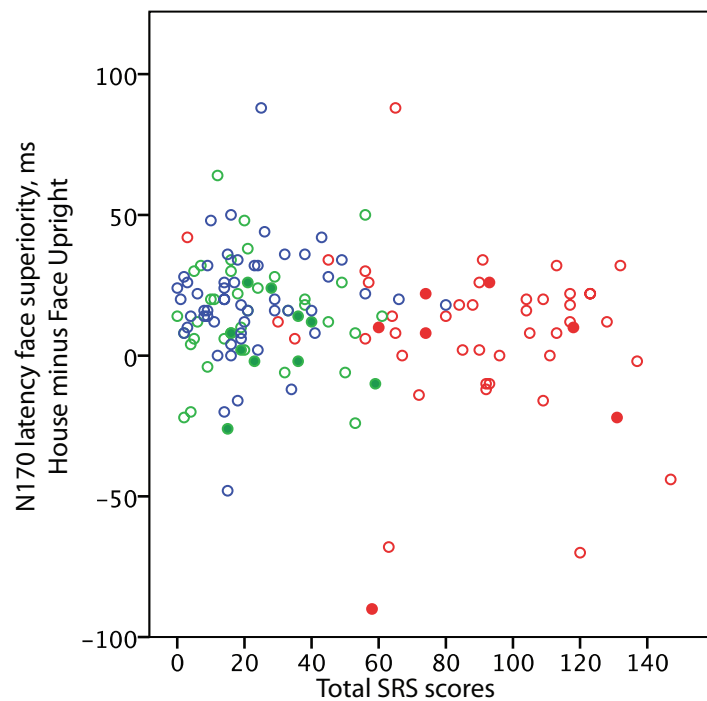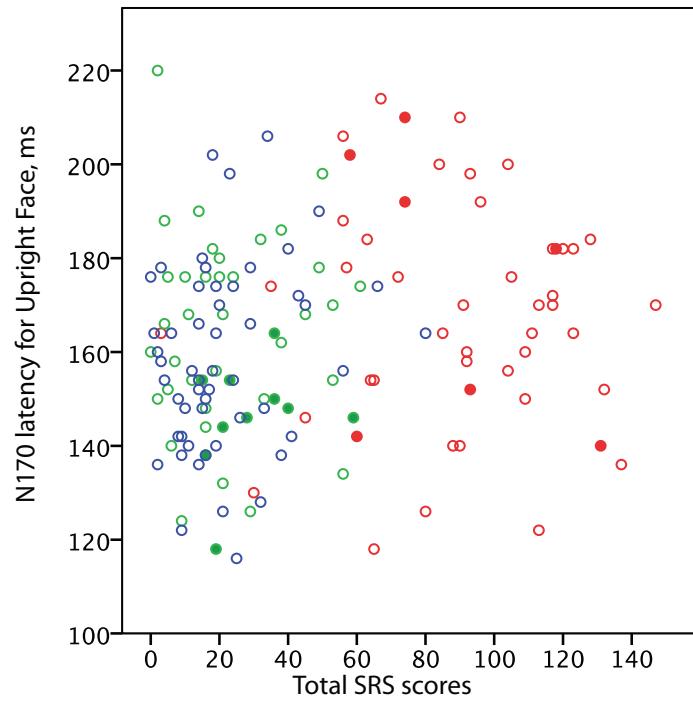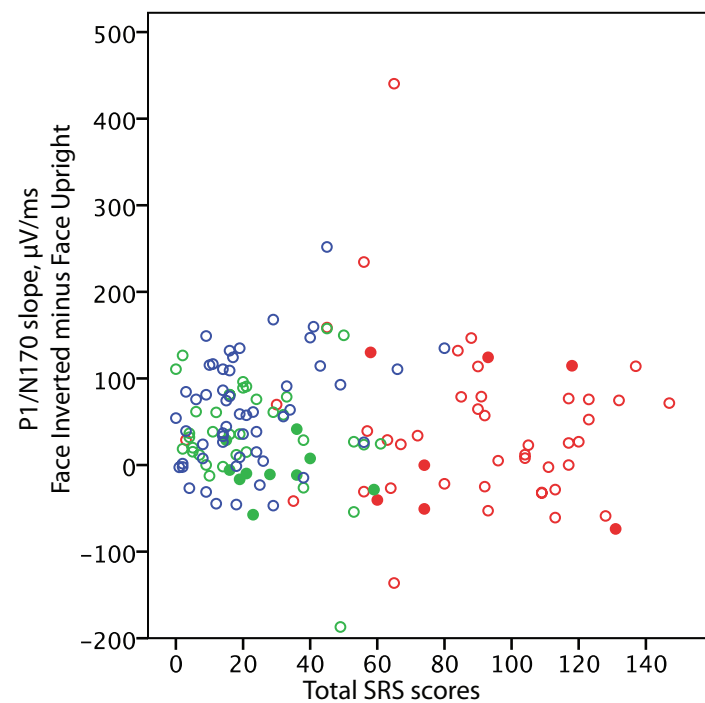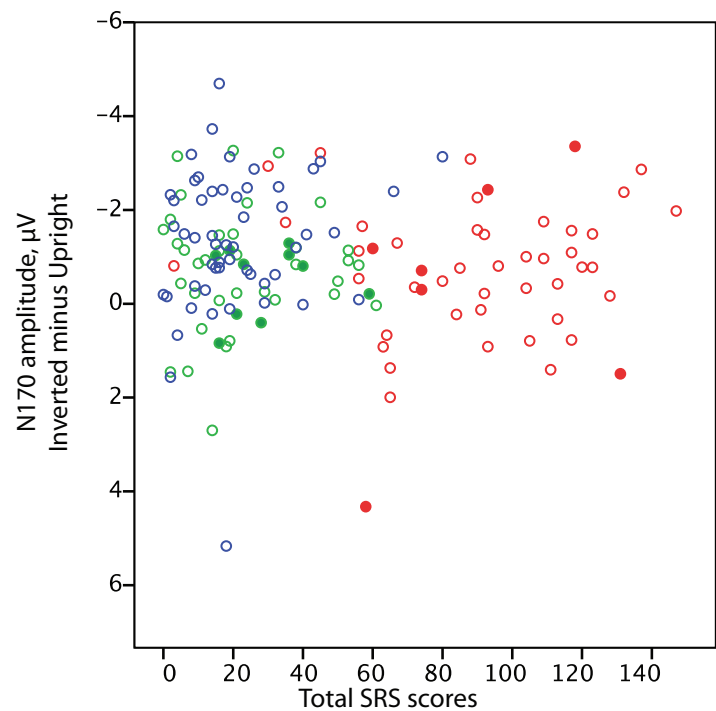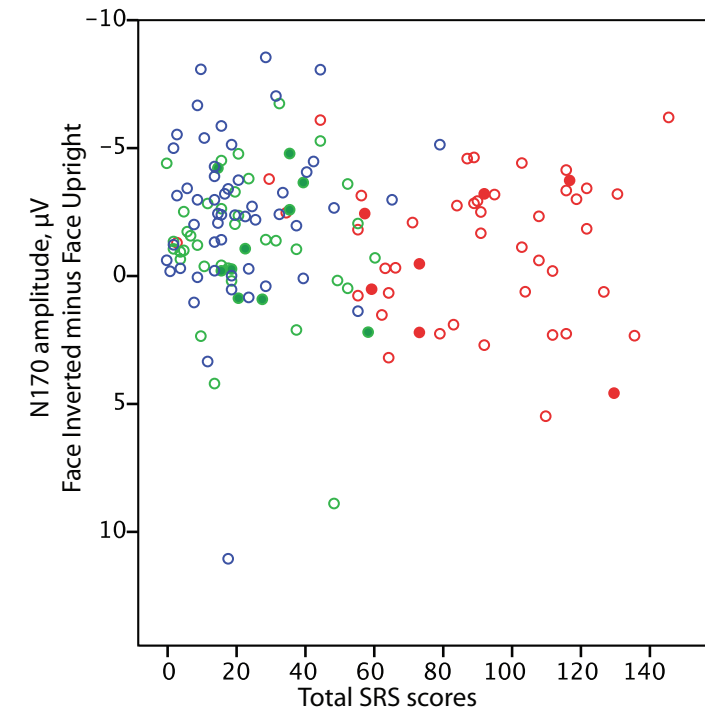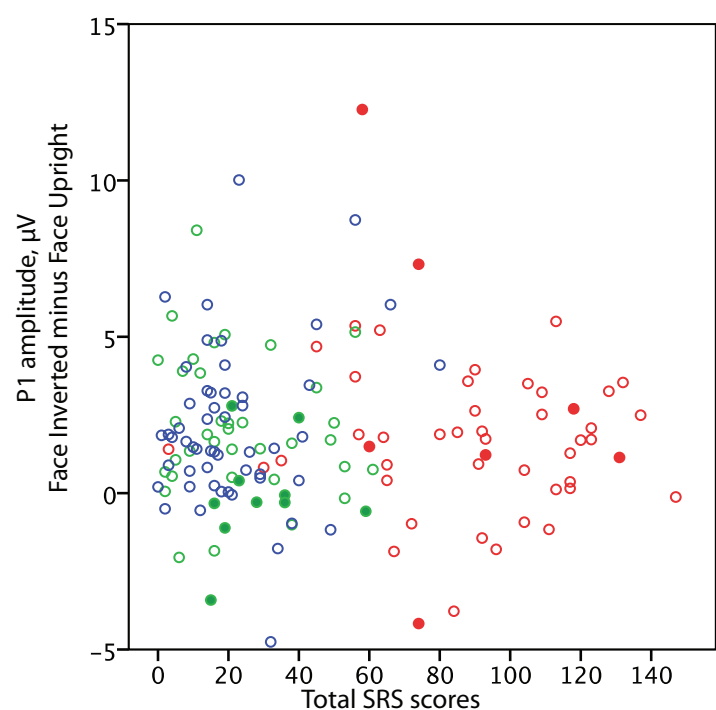

Supplement: Supplementary file 4 — Contains scatterplots depicting the (lack of) relationship between autistic trait severity measured by the Social Responsiveness Scale (SRS, X axis) and ERP contrasts of interest obtained with vertex (Cz) reference (Y axis): N170 latency for upright face stimuli (A), face superiority effect on N170 latency (B), face inversion effect on N170 amplitude (C), and P1 amplitude (D). Each dot represents an individual subject. Group membership is coded by color: red filled circles indicate children with autistic disorder (299.0), empty red circles stand for PDD_NOS/Asperger (299.80), green empty circles denote unaffected siblings (US), and blue empty circles with unrelated controls (UC). In general, these figures illustrate the lack of significant correlations between the ERP effects of interest and SRS scores in any of the studied groups. (PDF 503 kb) [file 13229_2018_220_MOESM4_ESM.pdf]
