# Supplementary material for: Event-related potential (ERP) correlates of face processing in verbal children with autism spectrum disorders (ASD) and their first-degree relatives: a family study
Source: Mol Autism. 2018 Jul 5;9:41. doi: 10.1186/s13229-018-0220-x (PMC6034210; doi:10.1186/s13229-018-0220-x)
Supplement: Supplementary file 5 — Contains supplementary analysis performed for checking additional ERPs characteristics, underlying the main study hypotheses: N170 latency for faces, P1, and N170 amplitude inversion effects. Significant ASD vs TD difference in N170 latency for faces underlie reduced face superiority effect seen for ASD children, presented in Table 4 in the manuscript. The group differences for N170 amplitude inversion effect corresponds with those seen for general N170 inversion effect, represented in Table 4 of the manuscript. No significant group differences were observed for P1 amplitude inversion effect. (DOCX 200 kb) [file 13229_2018_220_MOESM5_ESM.docx]

ANOVA results for ASD/US and UC groups: Nose Reference (Bonferroni uncorrected)

|  | P1 latency | | | P1 amplitude | | | N170 latency | | | N170 amplitude | | | P1/N170 slope | | |
| --- | --- | --- | --- | --- | --- | --- | --- | --- | --- | --- | --- | --- | --- | --- | --- |
| Source | F | Sig. | η^2^ | F | Sig. | η^2^ | F | Sig. | η^2^ | F | Sig. | η^2^ | F | Sig. | η^2^ |
| Type | 52.988 | .000 | .282 | 20.084 | .000 | .130 | 35.664 | .000 | .209 | 160.958 | .000 | .544 | 216.091 | .000 | .615 |
| Type * Group | .116 | .891 | .002 | 3.501 | .033 | .049 | .658 | .520 | .010 | .070 | .933 | .001 | 1.688 | .189 | .024 |
|  |  |  |  |  |  |  |  |  |  |  |  |  |  |  |  |
| Orientation | 19.449 | .000 | .126 | 13.818 | .000 | .093 | 20.162 | .000 | .130 | 41.789 | .000 | .236 | 108.381 | .000 | .445 |
| Orientation * Group | .179 | .836 | .003 | 1.578 | .210 | .023 | .128 | .880 | .002 | .041 | .960 | .001 | .612 | .544 | .009 |
|  |  |  |  |  |  |  |  |  |  |  |  |  |  |  |  |
| Hemisphere | 10.754 | .001 | .074 | .132 | .717 | .001 | .161 | .689 | .001 | 2.166 | .143 | .016 | 36.998 | .000 | .215 |
| Hemisphere * Group | .815 | .445 | .012 | 2.678 | .072 | .038 | .714 | .492 | .010 | .526 | .592 | .008 | .561 | .572 | .008 |
|  |  |  |  |  |  |  |  |  |  |  |  |  |  |  |  |
| Type * Orientation | 25.348 | .000 | .158 | 36.948 | .000 | .215 | .418 | .519 | .003 | .994 | .320 | .007 | 25.470 | .000 | .159 |
| Type * Orientation * Group | 1.152 | .319 | .017 | .149 | .862 | .002 | .102 | .903 | .002 | 2.009 | .138 | .029 | 1.331 | .268 | .019 |
|  |  |  |  |  |  |  |  |  |  |  |  |  |  |  |  |
| Type * Hemisphere | .548 | .460 | .004 | .598 | .441 | .004 | 10.014 | .002 | .069 | 9.041 | .003 | .063 | 6.010 | .015 | .043 |
| Type * Hemisphere * Group | .087 | .917 | .001 | 3.186 | .044 | .045 | .429 | .652 | .006 | .296 | .744 | .004 | 1.677 | .191 | .024 |
|  |  |  |  |  |  |  |  |  |  |  |  |  |  |  |  |
| Orientation * Hemisphere | .001 | .979 | .000 | 5.506 | .020 | .039 | .212 | .646 | .002 | 19.351 | .000 | .125 | 4.905 | .028 | .035 |
| Orientation * Hemisphere * Group | 2.230 | .112 | .032 | .264 | .769 | .004 | 4.005 | .020 | .056 | .371 | .691 | .005 | .425 | .654 | .006 |
|  |  |  |  |  |  |  |  |  |  |  |  |  |  |  |  |
| Type * Orientation * Hemisphere | 1.773 | .185 | .013 | 1.483 | .225 | .011 | .097 | .756 | .001 | .270 | .604 | .002 | .623 | .431 | .005 |
| Type * Orientation * Hemisphere * Group | .358 | .700 | .005 | .111 | .895 | .002 | 2.049 | .133 | .029 | .332 | .718 | .005 | 1.257 | .288 | .018 |
| Group | 1.904 | .153 | .027 | 1.433 | .242 | .021 | .766 | .467 | .011 | .657 | .520 | .010 | .721 | .488 | .011 |

ANOVA results for ASD/US and UC groups: REST Reference (Bonferroni uncorrected)

|  | P1 latency | | | P1 amplitude | | | N170 latency | | | N170 amplitude | | | P1/N170 slope | | |
| --- | --- | --- | --- | --- | --- | --- | --- | --- | --- | --- | --- | --- | --- | --- | --- |
| Source | F | Sig. | η^2^ | F | Sig. | η^2^ | F | Sig. | η^2^ | F | Sig. | η^2^ | F | Sig. | η^2^ |
| Type | 29.087 | .000 | .177 | 27.480 | .000 | .169 | 54.913 | .000 | .289 | 183.774 | .000 | .577 | 172.987 | .000 | .562 |
| Type * Group | .057 | .945 | .001 | 1.831 | .164 | .026 | 3.438 | .035 | .048 | .635 | .531 | .009 | 1.671 | .192 | .024 |
|  |  |  |  |  |  |  |  |  |  |  |  |  |  |  |  |
| Orientation | 22.447 | .000 | .143 | 25.175 | .000 | .157 | 15.877 | .000 | .105 | 73.100 | .000 | .351 | 122.859 | .000 | .476 |
| Orientation * Group | .971 | .381 | .014 | .372 | .690 | .005 | 1.103 | .335 | .016 | 1.398 | .251 | .020 | 3.294 | .040 | .047 |
|  |  |  |  |  |  |  |  |  |  |  |  |  |  |  |  |
| Hemisphere | 14.110 | .000 | .095 | .526 | .470 | .004 | .016 | .900 | .000 | 1.625 | .205 | .012 | 37.957 | .000 | .219 |
| Hemisphere * Group | .111 | .895 | .002 | 1.940 | .148 | .028 | .643 | .527 | .009 | .352 | .704 | .005 | .839 | .435 | .012 |
|  |  |  |  |  |  |  |  |  |  |  |  |  |  |  |  |
| Type * Orientation | 12.466 | .001 | .085 | 59.266 | .000 | .305 | .748 | .389 | .006 | .348 | .556 | .003 | 29.872 | .000 | .181 |
| Type * Orientation * Group | 1.991 | .141 | .029 | .419 | .659 | .006 | 1.999 | .139 | .029 | 1.372 | .257 | .020 | 2.774 | .066 | .039 |
|  |  |  |  |  |  |  |  |  |  |  |  |  |  |  |  |
| Type * Hemisphere | .089 | .766 | .001 | 1.617 | .206 | .012 | 14.458 | .000 | .097 | 10.151 | .002 | .070 | 12.390 | .001 | .084 |
| Type * Hemisphere * Group | .013 | .988 | .000 | 1.766 | .175 | .025 | .027 | .973 | .000 | .198 | .821 | .003 | 1.536 | .219 | .022 |
|  |  |  |  |  |  |  |  |  |  |  |  |  |  |  |  |
| Orientation * Hemisphere | .569 | .452 | .004 | 3.781 | .054 | .027 | 1.892 | .171 | .014 | 18.567 | .000 | .121 | 2.882 | .092 | .021 |
| Orientation * Hemisphere * Group | .832 | .437 | .012 | .085 | .918 | .001 | .759 | .470 | .011 | .423 | .656 | .006 | .278 | .757 | .004 |
|  |  |  |  |  |  |  |  |  |  |  |  |  |  |  |  |
| Type * Orientation * Hemisphere | .000 | .993 | .000 | .508 | .477 | .004 | .030 | .862 | .000 | .612 | .435 | .005 | 1.205 | .274 | .009 |
| Type * Orientation * Hemisphere * Group | .421 | .657 | .006 | .531 | .590 | .008 | .196 | .822 | .003 | .181 | .834 | .003 | 1.980 | .142 | .028 |
| Group | 1.643 | .197 | .024 | 1.019 | .364 | .015 | .626 | .536 | .009 | .734 | .482 | .011 | .684 | .506 | .010 |

ANOVA results for ASD/US and UC groups: Average Reference (Bonferroni uncorrected)

|  | P1 latency | | | P1 amplitude | | | N170 latency | | | N170 amplitude | | | P1/N170 slope | | |
| --- | --- | --- | --- | --- | --- | --- | --- | --- | --- | --- | --- | --- | --- | --- | --- |
| Source | F | Sig. | η^2^ | F | Sig. | η^2^ | F | Sig. | η^2^ | F | Sig. | η^2^ | F | Sig. | η^2^ |
| Type | 71.465 | .000 | .346 | 12.544 | .001 | .085 | 83.190 | .000 | .381 | 373.861 | .000 | .735 | 265.314 | .000 | .663 |
| Type * Group | .675 | .511 | .010 | 2.934 | .057 | .042 | 2.571 | .080 | .037 | .714 | .492 | .010 | .731 | .483 | .011 |
|  |  |  |  |  |  |  |  |  |  |  |  |  |  |  |  |
| Orientation | 16.876 | .000 | .111 | 18.998 | .000 | .123 | 27.685 | .000 | .170 | 55.306 | .000 | .291 | 87.555 | .000 | .393 |
| Orientation * Group | .736 | .481 | .011 | .357 | .701 | .005 | .537 | .586 | .008 | 2.788 | .065 | .040 | 2.602 | .078 | .037 |
|  |  |  |  |  |  |  |  |  |  |  |  |  |  |  |  |
| Hemisphere | 12.356 | .001 | .084 | .758 | .385 | .006 | .113 | .737 | .001 | 2.425 | .122 | .018 | 40.793 | .000 | .232 |
| Hemisphere * Group | .449 | .639 | .007 | 2.235 | .111 | .032 | .221 | .802 | .003 | .644 | .527 | .009 | .870 | .421 | .013 |
|  |  |  |  |  |  |  |  |  |  |  |  |  |  |  |  |
| Type * Orientation | 20.885 | .000 | .134 | 67.632 | .000 | .334 | 4.097 | .045 | .029 | .719 | .398 | .005 | 18.453 | .000 | .120 |
| Type * Orientation * Group | 2.054 | .132 | .030 | .003 | .997 | .000 | .029 | .972 | .000 | .751 | .474 | .011 | 1.829 | .164 | .026 |
|  |  |  |  |  |  |  |  |  |  |  |  |  |  |  |  |
| Type * Hemisphere | 1.886 | .172 | .014 | 5.624 | .019 | .040 | 8.144 | .005 | .057 | 12.275 | .001 | .083 | 18.535 | .000 | .121 |
| Type * Hemisphere * Group | .734 | .482 | .011 | .468 | .627 | .007 | 1.431 | .243 | .021 | .323 | .725 | .005 | 1.533 | .220 | .022 |
|  |  |  |  |  |  |  |  |  |  |  |  |  |  |  |  |
| Orientation * Hemisphere | .102 | .750 | .001 | 5.113 | .025 | .036 | 4.112 | .045 | .030 | 23.166 | .000 | .146 | 3.975 | .048 | .029 |
| Orientation * Hemisphere * Group | .643 | .527 | .009 | .043 | .958 | .001 | .663 | .517 | .010 | .273 | .761 | .004 | .976 | .380 | .014 |
|  |  |  |  |  |  |  |  |  |  |  |  |  |  |  |  |
| Type * Orientation * Hemisphere | .200 | .656 | .001 | 1.419 | .236 | .010 | 1.967 | .163 | .014 | .518 | .473 | .004 | .594 | .442 | .004 |
| Type * Orientation * Hemisphere * Group | .287 | .751 | .004 | .015 | .985 | .000 | 1.594 | .207 | .023 | .670 | .513 | .010 | .755 | .472 | .011 |
| Group | 1.729 | .181 | .025 | 1.251 | .290 | .018 | .986 | .376 | .014 | .764 | .468 | .011 | .239 | .788 | .004 |

ANOVA results for ASD/US and UC groups: Vertex Reference (Bonferroni uncorrected)

|  | P1 latency | | | P1 amplitude | | | N170 latency | | | N170 amplitude | | | P1/N170 slope | | |
| --- | --- | --- | --- | --- | --- | --- | --- | --- | --- | --- | --- | --- | --- | --- | --- |
| Source | F | Sig. | η^2^ | F | Sig. | η^2^ | F | Sig. | η^2^ | F | Sig. | η^2^ | F | Sig. | η^2^ |
| Type | 137.684 | .000 | .505 | 4.634 | .033 | .033 | 62.461 | .000 | .316 | 377.614 | .000 | .737 | 297.431 | .000 | .688 |
| Type * Group | 1.841 | .163 | .027 | .499 | .608 | .007 | 2.341 | .100 | .034 | 2.753 | .067 | .039 | .158 | .854 | .002 |
|  |  |  |  |  |  |  |  |  |  |  |  |  |  |  |  |
| Orientation | 18.563 | .000 | .121 | 20.732 | .000 | .133 | 19.128 | .000 | .124 | 46.012 | .000 | .254 | 62.365 | .000 | .316 |
| Orientation * Group | .478 | .621 | .007 | 1.388 | .253 | .020 | 1.995 | .140 | .029 | 5.142 | .007 | .071 | 2.455 | .090 | .035 |
|  |  |  |  |  |  |  |  |  |  |  |  |  |  |  |  |
| Hemisphere | 7.904 | .006 | .055 | .553 | .458 | .004 | .095 | .759 | .001 | 2.541 | .113 | .018 | 37.128 | .000 | .216 |
| Hemisphere * Group | .508 | .603 | .007 | 3.790 | .025 | .053 | .105 | .901 | .002 | .852 | .429 | .012 | .974 | .380 | .014 |
|  |  |  |  |  |  |  |  |  |  |  |  |  |  |  |  |
| Type * Orientation | 13.454 | .000 | .091 | 36.117 | .000 | .211 | 3.704 | .056 | .027 | 2.622 | .108 | .019 | 19.803 | .000 | .128 |
| Type * Orientation * Group | .264 | .769 | .004 | 1.135 | .324 | .017 | .363 | .696 | .005 | .358 | .700 | .005 | .371 | .691 | .005 |
|  |  |  |  |  |  |  |  |  |  |  |  |  |  |  |  |
| Type * Hemisphere | 1.766 | .186 | .013 | 3.347 | .070 | .024 | 1.091 | .298 | .008 | 13.363 | .000 | .090 | 12.993 | .000 | .088 |
| Type * Hemisphere * Group | .188 | .829 | .003 | 1.546 | .217 | .022 | .895 | .411 | .013 | .502 | .607 | .007 | .346 | .708 | .005 |
|  |  |  |  |  |  |  |  |  |  |  |  |  |  |  |  |
| Orientation * Hemisphere | 2.227 | .138 | .016 | 7.216 | .008 | .051 | 2.060 | .154 | .015 | 30.012 | .000 | .182 | 3.458 | .065 | .025 |
| Orientation * Hemisphere * Group | .350 | .706 | .005 | .207 | .813 | .003 | .302 | .740 | .004 | .016 | .984 | .000 | .209 | .811 | .003 |
|  |  |  |  |  |  |  |  |  |  |  |  |  |  |  |  |
| Type * Orientation * Hemisphere | 6.652 | .011 | .047 | 5.696 | .018 | .040 | 1.278 | .260 | .009 | .142 | .707 | .001 | 1.821 | .179 | .013 |
| Type * Orientation * Hemisphere * Group | .476 | .622 | .007 | .151 | .860 | .002 | .672 | .513 | .010 | .600 | .550 | .009 | 1.361 | .260 | .020 |
| Group | 1.153 | .319 | .017 | 1.078 | .343 | .016 | 1.232 | .295 | .018 | .747 | .476 | .011 | .206 | .814 | .003 |

ANOVA results for UF group: Nose Reference

|  | P1 latency | | | P1 amplitude | | | N170 latency | | | N170 amplitude | | | P1/N170 slope | | |
| --- | --- | --- | --- | --- | --- | --- | --- | --- | --- | --- | --- | --- | --- | --- | --- |
| Source | F | Sig. | η^2^ | F | Sig. | η^2^ | F | Sig. | η^2^ | F | Sig. | η^2^ | F | Sig. | η^2^ |
| Type | 2.591 | .126 | .132 | 11.447 | .004 | .402 | 1.446 | .246 | .078 | 28.224 | .000 | .624 | 37.331 | .000 | .687 |
| Orientation | 15.959 | .001 | .484 | .006 | .937 | .000 | 36.095 | .000 | .680 | 2.380 | .141 | .123 | .793 | .386 | .045 |
| Hemisphere | .314 | .583 | .018 | .738 | .402 | .042 | .042 | .839 | .002 | .562 | .464 | .032 | .994 | .333 | .055 |
|  |  |  |  |  |  |  |  |  |  |  |  |  |  |  |  |
| Type * Orientation | .523 | .480 | .030 | 1.874 | .189 | .099 | 2.626 | .124 | .134 | .172 | .684 | .010 | 4.913 | .041 | .224 |
| Type * Hemisphere | .544 | .471 | .031 | .423 | .524 | .024 | .115 | .739 | .007 | .750 | .399 | .042 | .495 | .491 | .028 |
| Orientation * Hemisphere | 1.510 | .236 | .082 | .046 | .833 | .003 | .188 | .670 | .011 | .081 | .780 | .005 | .402 | .535 | .023 |
|  |  |  |  |  |  |  |  |  |  |  |  |  |  |  |  |
| Type * Orientation * Hemisphere | 3.703 | .071 | .179 | .277 | .606 | .016 | .670 | .424 | .038 | .641 | .435 | .036 | .501 | .489 | .029 |

ANOVA results for UF group: REST Reference

|  | P1 latency | | | P1 amplitude | | | N170 latency | | | N170 amplitude | | | P1/N170 slope | | |
| --- | --- | --- | --- | --- | --- | --- | --- | --- | --- | --- | --- | --- | --- | --- | --- |
| Source | F | Sig. | η^2^ | F | Sig. | η^2^ | F | Sig. | η^2^ | F | Sig. | η^2^ | F | Sig. | η^2^ |
| Type | 2.778 | .114 | .140 | 18.213 | .001 | .517 | 5.609 | .030 | .248 | 39.657 | .000 | .700 | 27.994 | .000 | .622 |
| Orientation | 12.978 | .002 | .433 | .121 | .733 | .007 | 32.561 | .000 | .657 | 5.427 | .032 | .242 | .928 | .349 | .052 |
| Hemisphere | .140 | .713 | .008 | 1.171 | .294 | .064 | .392 | .540 | .023 | .866 | .365 | .048 | 1.984 | .177 | .105 |
|  |  |  |  |  |  |  |  |  |  |  |  |  |  |  |  |
| Type * Orientation | 2.678 | .120 | .136 | .799 | .384 | .045 | 5.827 | .027 | .255 | 2.428 | .138 | .125 | 5.557 | .031 | .246 |
| Type * Hemisphere | .061 | .807 | .004 | .451 | .511 | .026 | .016 | .902 | .001 | .801 | .383 | .045 | .037 | .851 | .002 |
| Orientation * Hemisphere | 1.481 | .240 | .080 | .114 | .740 | .007 | .429 | .521 | .025 | .045 | .835 | .003 | 1.274 | .275 | .070 |
|  |  |  |  |  |  |  |  |  |  |  |  |  |  |  |  |
| Type * Orientation * Hemisphere | 5.471 | .032 | .243 | .938 | .346 | .052 | .158 | .696 | .009 | .779 | .390 | .044 | .900 | .356 | .050 |

ANOVA results for UF group: Average Reference

|  | P1 latency | | | P1 amplitude | | | N170 latency | | | N170 amplitude | | | P1/N170 slope | | |
| --- | --- | --- | --- | --- | --- | --- | --- | --- | --- | --- | --- | --- | --- | --- | --- |
| Source | F | Sig. | η^2^ | F | Sig. | η^2^ | F | Sig. | η^2^ | F | Sig. | η^2^ | F | Sig. | η^2^ |
| Type | 2.206 | .156 | .115 | 24.048 | .000 | .586 | 11.429 | .004 | .402 | 61.829 | .000 | .784 | 67.135 | .000 | .798 |
| Orientation | 10.579 | .005 | .384 | .005 | .946 | .000 | 13.826 | .002 | .449 | 16.114 | .001 | .487 | .232 | .636 | .013 |
| Hemisphere | 2.268 | .150 | .118 | .939 | .346 | .052 | 1.366 | .259 | .074 | .282 | .602 | .016 | 1.895 | .186 | .100 |
|  |  |  |  |  |  |  |  |  |  |  |  |  |  |  |  |
| Type * Orientation | .002 | .964 | .000 | 1.678 | .213 | .090 | 3.274 | .088 | .161 | 7.934 | .012 | .318 | .296 | .593 | .017 |
| Type * Hemisphere | .016 | .902 | .001 | 1.064 | .317 | .059 | .238 | .632 | .014 | .737 | .403 | .042 | .418 | .527 | .024 |
| Orientation * Hemisphere | 2.849 | .110 | .144 | .073 | .790 | .004 | 1.587 | .225 | .085 | .143 | .710 | .008 | .317 | .581 | .018 |
|  |  |  |  |  |  |  |  |  |  |  |  |  |  |  |  |
| Type * Orientation * Hemisphere | .349 | .562 | .020 | .197 | .663 | .011 | 1.733 | .206 | .093 | .910 | .354 | .051 | .675 | .423 | .038 |

ANOVA results for UF group: Vertex Reference

|  | P1 latency | | | P1 amplitude | | | N170 latency | | | N170 amplitude | | | P1/N170 slope | | |
| --- | --- | --- | --- | --- | --- | --- | --- | --- | --- | --- | --- | --- | --- | --- | --- |
| Source | F | Sig. | η^2^ | F | Sig. | η^2^ | F | Sig. | η^2^ | F | Sig. | η^2^ | F | Sig. | η^2^ |
| Type | .189 | .670 | .011 | 23.882 | .000 | .584 | 5.387 | .033 | .241 | 73.109 | .000 | .811 | 59.021 | .000 | .776 |
| Orientation | 7.085 | .016 | .294 | .297 | .593 | .017 | 24.900 | .000 | .594 | 17.833 | .001 | .512 | .039 | .845 | .002 |
| Hemisphere | .120 | .734 | .007 | 1.164 | .296 | .064 | 1.061 | .317 | .059 | .199 | .661 | .012 | 1.054 | .319 | .058 |
|  |  |  |  |  |  |  |  |  |  |  |  |  |  |  |  |
| Type * Orientation | .410 | .531 | .024 | .263 | .615 | .015 | 1.499 | .238 | .081 | 9.889 | .006 | .368 | .461 | .506 | .026 |
| Type * Hemisphere | .421 | .525 | .024 | 1.259 | .277 | .069 | .896 | .357 | .050 | .841 | .372 | .047 | .106 | .749 | .006 |
| Orientation * Hemisphere | 1.372 | .258 | .075 | .007 | .935 | .000 | 1.115 | .306 | .062 | .068 | .797 | .004 | .135 | .718 | .008 |
|  |  |  |  |  |  |  |  |  |  |  |  |  |  |  |  |
| Type * Orientation * Hemisphere | 10.478 | .005 | .381 | .001 | .972 | .000 | 1.061 | .317 | .059 | .862 | .366 | .048 | 2.005 | .175 | .105 |
