# Supplementary material for: Event-related potential (ERP) correlates of face processing in verbal children with autism spectrum disorders (ASD) and their first-degree relatives: a family study
Source: Mol Autism. 2018 Jul 5;9:41. doi: 10.1186/s13229-018-0220-x (PMC6034210; doi:10.1186/s13229-018-0220-x)
Supplement: Supplementary file 6 — Contains discussion of potential moderating and confounding factors that may contribute to the observed discrepancy between results of our and some previous studies. Among considered factors are age, gender, low IQ, and ADHD subjects in our ASD group, attention to stimuli, and reference schemes. (DOC 36 kb) [file 13229_2018_220_MOESM6_ESM.doc]

Detailed discussion of potential moderating and confounding factors

Age. Our main sample consisted of individuals in the age range between 12 and 21 years and thus might have included individuals in whom the face recognition system was not ully developed [1, 2]. This fact may contribute to the differences between our results and those reported previously in adults. In particular, the reduced face inversion effect on P1/N170 slope has been reported only for adults with ASD [3]. In our study the only adult group was the group of unaffected fathers of ASD probands and no significant face inversion effect on P1/N170 slope was observed in this group of UF, however, the interpretation of this negative finding is hindered by the lack of a comparison sample of age-matched controls (Table 3, 4). Therefore, it cannot be excluded that face inversion effect on P1/N170 slope might reliably differentiate adult individuals with ASD from controls.

Conversely, the lack of difference between unaffected siblings of ASD probands and unrelated controls in face over object superiority effect in N170 timing is unlikely to be specific to the child population. Our adult sample of unaffected fathers of ASD probands exhibited a significant face over object superiority effect on N170 timing of similar size to previously reported effects in an unrelated control group [4].

ERP studies in children are particularly challenging. Achieving adequate signal-to-noise ratio may be more difficult in children than adults (note that about half of the subjects in [5] were excluded from the analysis due to low signal-to-noise ratio). As noted above, face recognition skills are not fully developed until adulthood, and both N170 and P1 components are significantly affected by age in ways that may or may not be specific to elicitation by face stimuli [6]. Moreover, prior to age 12, N170 shows a double peak [6]; it is unknown which peak should be chosen for consistent comparison with older groups. Children also have broader individual variability of ERP measures than adults [7]. All of these factors could influence between-group comparisons. Our study mitigated some of these issues by restricting the sample to older children (age 12 and over).

Gender. The inclusion of females in previous studies might have contributed to the discrepancies between those studies and present results obtained exclusively in male population. For example, face-superiority effect on N170 latency was absent in the study of Dawson and colleagues [4] including mostly female parents of children with ASD, but was typical in our sample of unaffected fathers of two or more children with ASD. In a recent study, the face inversion effect on N170 amplitude was significantly smaller in females than in males with ASD [9]. Thus, face-sensitive ERP characteristics might be more severely affected in females than males with ASD. Such gender difference may be related to more profound impact of ASD on females. The rate of ASD in females is 4 times lower than that in males, suggesting compensatory mechanisms protecting females from phenotypic manifestations of inherited ASD liability [8], and indicating that ASD-affected females either lack such mechanisms or have highly elevated genetic burden for ASD.

Low IQ and ADHD. The exclusion of subjects with low IQ and ADHD did not alter the findings for P1 and N170 components.

Attention to the stimuli. A lack of attention-to-task in general and to the eyes area of the face, in particular, can influence the N170/P1 components [10-14]. Furthermore, adding a cross hair to direct attention to the stimuli led to an increase of fusiform activity and abolished differences between ASD and controls in a functional magnetic resonance imaging (fMRI) study [15]. Another fMRI study [61] reported that activation of the fusiform face area in children with autism was predicted by the amount of time during which the subjects fixated on the eyes region of the face. Therefore, insufficient task engagement may confound ERP differences between individuals and controls. Due to their lack of interest to facial stimuli individuals may not be fully engaged in the task, in particular, they may fail to attend to the eyes region of the face.

It seems unlikely that our results are related to attention issues, as our study implemented a strategy to draw the subjects’ attention to the eyes area: all facial images were standardized so that the eyes area was aligned with the center of the screen, and all subjects were engaged in an embedded attention task to screen out those unable to sustain fixation. This design not only provides an efficient means to draw the subject’s attention to the eyes region of the face, but also provides objective control over the subject’s compliance with the instruction and allows for identification and exclusion of subjects with poor task engagement.

The lack of attention to eyes might trigger the reduction of face over object superiority effect on N170 timing in parents of ASD children, as reported by Dawson and colleagues [4] as no fixation cross was utilized in that study. However, adding the fixation cross did not eliminate the difference between ASD and UC groups in our study or others [5, 7].

Clinical control. Many other psychiatric conditions including schizophrenia, depression, anxiety, and even environmental stressors or aggression have been observed to contribute to atypical face processing [17, 18]. The unaffected control group in our study included siblings of children with a variety of diagnosed psychiatric conditions other than ASD. Since most of previous studies of face processing in ASD did not include such clinical controls, the current study design set a higher bar for the detection of group differences relative to previous studies. On the other hand, by using controls who may be carriers of predisposition factors to other psychiatric disorders, our search for group differences construed as restricted to those abnormalities in face processing that are relatively specific to ASD and are not shared with other disorders.

References

1. Ellis HD. The development of face processing skills. Philos Trans R Soc Lond B Biol Sci. 1992;335(1273):105-11. doi:10.1098/rstb.1992.0013.

2. Chung MS, Thomson DM. Development of face recognition. Br J Psychol. 1995;86 ( Pt 1):55-87.

3. Webb SJ, Merkle K, Murias M, Richards T, Aylward E, Dawson G. ERP responses differentiate inverted but not upright face processing in adults with ASD. Soc Cogn Affect Neurosci. 2012;7(5):578-87. doi:nsp002 [pii] 10.1093/scan/nsp002.

4. Dawson G, Webb SJ, Wijsman E, Schellenberg G, Estes A, Munson J et al. Neurocognitive and electrophysiological evidence of altered face processing in parents of children with autism: implications for a model of abnormal development of social brain circuitry in autism. Dev Psychopathol. 2005;17(3):679-97. doi:S0954579405050327 [pii] 10.1017/S0954579405050327.

5. McPartland JC, Wu J, Bailey CA, Mayes LC, Schultz RT, Klin A. Atypical neural specialization for social percepts in autism spectrum disorder. Soc Neurosci. 2011;6(5-6):436-51. doi:10.1080/17470919.2011.586880.

6. Taylor MJ, Batty M, Itier RJ. The faces of development: a review of early face processing over childhood. J Cogn Neurosci. 2004;16(8):1426-42. doi:10.1162/0898929042304732.

7. O'Connor K, Hamm JP, Kirk IJ. Neurophysiological responses to face, facial regions and objects in adults with Asperger's syndrome: an ERP investigation. Int J Psychophysiol. 2007;63(3):283-93. doi:S0167-8760(06)00320-5 [pii] 10.1016/j.ijpsycho.2006.12.001.

8. Constantino JN, Charman T. Gender bias, female resilience, and the sex ratio in autism. J Am Acad Child Adolesc Psychiatry. 2012;51(8):756-8. doi:S0890-8567(12)00411-X [pii]10.1016/j.jaac.2012.05.017.

9. Coffman MC, Anderson LC, Naples AJ, McPartland JC. Sex differences in social perception in children with ASD. J. Autism Dev. Disord. 2015;45:589–99. doi: 10.1007/s10803-013-2006-5.

10. Eimer M. Attentional modulations of event-related brain potentials sensitive to faces. Cogn Neuropsychol. 2000;17(1):103-16. doi:713751850 [pii] 10.1080/026432900380517.

11. Furey ML, Tanskanen T, Beauchamp MS, Avikainen S, Uutela K, Hari R et al. Dissociation of face-selective cortical responses by attention. Proc Natl Acad Sci U S A. 2006;103(4):1065-70. doi:0510124103 [pii] 10.1073/pnas.0510124103.

12. Churches O, Wheelwright S, Baron-Cohen S, Ring H. The N170 is not modulated by attention in autism spectrum conditions. Neuroreport. 2010;21(6):399-403.

13. Nasr S. Differential impact of attention on the early and late categorization related human brain potentials. J Vis. 2010;10(11):18. doi:10.11.18 [pii] 10.1167/10.11.18.

14. Wijers AA, Banis S. Foveal and parafoveal spatial attention and its impact on the processing of facial expression: an ERP study. Clin Neurophysiol. 2012;123(3):513-26. doi:S1388-2457(11)00543-8 [pii] 10.1016/j.clinph.2011.07.040.

15. Hadjikhani N, Chabris CF, Joseph RM, Clark J, McGrath L, Aharon I et al. Early visual cortex organization in autism: an fMRI study. Neuroreport. 2004;15(2):267-70.

16. Dalton KM, Nacewicz BM, Johnstone T, Schaefer HS, Gernsbacher MA, Goldsmith HH et al. Gaze fixation and the neural circuitry of face processing in autism. Nat Neurosci. 2005;8(4):519-26. doi:nn1421 [pii] 10.1038/nn1421.

17. Feuerriegel D, Churches O, Hofmann J, Keage HA. The N170 and face perception in psychiatric and neurological disorders: A systematic review. Clin Neurophysiol. 2015;126(6):1141-58. doi:10.1016/j.clinph.2014.09.015.

18. Hole G, Bourne V. Face Processing: Psychological, Neuropsychological, and Applied Perspectives. OUP Oxford; 2010.
